# Precordial Vibrations: A Review of Wearable Systems, Signal Processing Techniques, and Main Applications

**DOI:** 10.3390/s22155805

**Published:** 2022-08-03

**Authors:** Francesca Santucci, Daniela Lo Presti, Carlo Massaroni, Emiliano Schena, Roberto Setola

**Affiliations:** 1 Unit of Automatic Control, Departmental Faculty of Engineering, Università Campus Bio-Medico di Roma, Via Alvaro del Portillo 21, 00128 Rome, Italy; r.setola@unicampus.it; 2 Unit of Measurements and Biomedical Instrumentation, Departmental Faculty of Engineering, Università Campus Bio-Medico di Roma, Via Alvaro del Portillo 21, 00128 Rome, Italy; d.lopresti@unicampus.it (D.L.P.); c.massaroni@unicampus.it (C.M.); e.schena@unicampus.it (E.S.)

**Keywords:** precordial vibrations, wearable systems, seismocardiography (SCG), gyrocardiography, fiber Bragg grating sensors, SCG annotation, SCG processing techniques, SCG fiducial points, SCG applications, machine learning

## Abstract

Recently, the ever-growing interest in the continuous monitoring of heart function in out-of-laboratory settings for an early diagnosis of cardiovascular diseases has led to the investigation of innovative methods for cardiac monitoring. Among others, wearables recording seismic waves induced on the chest surface by the mechanical activity of the heart are becoming popular. For what concerns wearable-based methods, cardiac vibrations can be recorded from the thorax in the form of acceleration, angular velocity, and/or displacement by means of accelerometers, gyroscopes, and fiber optic sensors, respectively. The present paper reviews the currently available wearables for measuring precordial vibrations. The focus is on sensor technology and signal processing techniques for the extraction of the parameters of interest. Lastly, the explored application scenarios and experimental protocols with the relative influencing factors are discussed for each technique. The goal is to delve into these three fundamental aspects (i.e., wearable system, signal processing, and application scenario), which are mutually interrelated, to give a holistic view of the whole process, beyond the sensor aspect alone. The reader can gain a more complete picture of this context without disregarding any of these 3 aspects.

## 1. Introduction and Physiological Sources of Displacement Cardiography

Early detection of any abnormality that affects the physiological heart function has been shown to be crucial in reducing the burden of cardiovascular diseases (CVDs) on the healthcare system in terms of both economic loss (expected to be 47 trillion US dollars by 2030 [1]) and medical personnel overload (about 17.8 million global deaths per year [2]).

Several studies have demonstrated that monitoring physiological parameters and hemodynamics for heart failure (HF) patients can allow the early detection of worsening symptoms prior to an exacerbation [3]. For this reason, in recent years, there has been a growing interest in developing innovative solutions based on wearable systems, which enable monitoring the cardiovascular function outside of laboratory settings in ambulant subjects, allowing an early diagnosis and management of CVDs and their risk factors. Today, electrocardiogram (ECG) and photoplethysmography (PPG) are still the dominant cardiac monitoring techniques implementable in a wearable configuration. However, innovative methods are gaining growing attention for the evaluation of cardiac function in daily life, such as those which measure seismic waves produced on the thorax surface by the mechanical activity of the heart: the heart beats as a result of mechanical events triggered by electrical signals. Within a single cardiac cycle, the following mechanical phases can be identified:

- Isovolumetric ventricular contraction: at first, isovolumetric ventricular contraction causes ventricular pressure to rise above atrial pressure, forcing the atrioventricular (AV) valves to close. The continuing contraction with the valves closed increases ventricular pressure.
- Ventricular ejection: occurs when ventricular pressure rises above arterial pressure and the semilunar valves open. As blood is ejected into the arteries, ventricular volume decreases, and ventricles begin to repolarize and relax. Ventricular pressure decreases and contraction ends.
- Isovolumetric ventricular relaxation: repolarization of the ventricular muscle cells initiates isovolumetric ventricular relaxation. As the ventricles relax, pressure in the ventricles drops and the semilunar valves close, preventing blood reflux. Valve closure produces a dicrotic wave on the aortic pressure curve. This isovolumetric relaxation makes pressure drop quickly.
- Passive ventricular filling: as all four chambers of the heart are relaxed and the AV valves open, passive ventricular filling starts. Atrial depolarization triggers atrial contraction and a new cardiac cycle begins.

Within each cardiac cycle, the contraction (i.e., systole) and relaxation (i.e., diastole) of helically oriented muscle fibers cause the longitudinal retraction of the left-ventricular base toward the apex, while valve closure and pumping of blood to the aorta generate a strong pressure shock [4]. The originated vibrations propagate by causing cyclic compressions and elongations of the nonhomogeneous and anisotropic tissues within the thorax. The linear viscoelastic behavior of the biological tissues is mainly responsible for dampening the propagation of vibrations from the heart to the thorax surface. As a result, these compression waves manifest themselves on the skin in the form of microscopic vibrations of amplitude 0.2–0.5 mm along the sagittal plane [5].

The mechanical vibrations involve four frequency bands: below 50 Hz, 150–200 Hz, 500–600 Hz, and 700–800 Hz [6]. These values depend on different cardiac mechanical events, such as the opening and closure of valves (i.e., mitral valve and aortic valve), blood flow, and heart wall deformation (i.e., compression/expansion along the long axis of the heart) due to heartbeat. More in detail, frequencies below 50 Hz are traceable to the cyclic motion of the contour of the heart, while all the frequencies above 50 Hz are related to the activity of the valves that regulate the resulting flow of blood across the chambers and its ejection into the vascular tree. These vibrations include inaudible (0.6–20 Hz) and audible (above 20 Hz) frequencies. 

Signals that result from cardiac vibrations have been investigated to determine their relationship with the mechanical events of the cardiac cycle. These signals have a potential utility in noninvasive cardiology, because they may allow detecting any defect in the heart valve functioning or in blood flow dynamics. Instrumentation used to selectively record cardiac mechanics from the chest surface belongs to the general category of displacement cardiography. For what concerns contact-based methods, cardiac vibrations can be recorded from the thorax as accelerations and angular velocities using magneto-inertial units (i.e., by accelerometers and gyroscopes) or as local deformations using strain sensors (especially, fiber Bragg gratings—FBGs) [7]. According to the measurand, signals can be distinguished into a seismocardiogram (SCG), which generally results from the linear acceleration component of precordial motion, and the gyrocardiogram (GCG), which represents the rotational component of precordial motion [8,9]. 

Currently, the most well-established technique is seismocardiography, which is performed by placing ultralow-frequency accelerometers onto the subject’s chest wall. The SCG signal was first discovered by Baevskii et al. in 1964 and introduced into clinical practice by Salerno and Zanetti in the 1990s [10]. Over the years, advances in sensor technologies and signal processing led to more portable and wireless sensors for SCG acquisition in various scenarios, helping to deepen the knowledge on the clinical power of this signal. Another interesting approach was introduced in 2017 and consists in the use of MEMS gyroscopes to record the three-dimensional angular velocity and displacement of the thorax associated with cardiac activity [9]. Although the history of gyrocardiography is brief compared to that of seismocardiography, this technique may provide additional understandings about the mechanical aspects of the cardiac cycle. Indeed, the GCG signal has a higher signal-to-noise ratio than the SCG signal; hence, it could provide novel insights into cardiac fiducial points, higher fidelity for certain types of motion artefacts, and a more reliable heart rate (HR) detection when using kinetic energy envelopes [9,11].

In recent years, one of the most promising technological solutions for monitoring cardiac mechanics is represented by FBG sensors. These sensors are placed on the surface of the chest to detect heart-induced local deformations. In the literature, works commonly refer to the cardiac FBG signal as an SCG when detected on the chest. The FBG-based systems, based on fiber optic technology, have the great advantage of working in a harsh environment, such as in the presence of strong electromagnetic fields (e.g., during magnetic resonance scans), where the use of electronic sensors is forbidden [12,13].

In order to switch from the use of mechanical cardiac signals (e.g., SCG and GCG) in research fields to their use in clinics, it is fundamental to quantify the signal waveforms. For instance, it is important to determine the temporal shift between each fiducial point and the successive one to determine the spatial distribution of fiducial points on the SCG waveform without the need for any additional reference signal [14].

This paper reviews the measuring systems used for noninvasive recording of cardiac mechanics. Since methods for monitoring cardiac mechanics are gaining increasing attention, we have focused on these techniques excluding wearable-based methods that rely on the measurement of different physical quantities (e.g., ECG and PPG). Firstly, we focus on the most popular technique: accelerometers for SCG signal acquisition. Then, we describe the two main novel approaches proposed in the literature: gyroscopes for GCG measurements and FBG-based systems for detecting chest wall deformations. For each category, we focus on their working principle as wearable systems and pay particular attention to their metrological aspects. The fundamental hardware components embedded into the wearable devices are illustrated by detailing the sensing element, the transmission, and the storage units. Then, the most popular signal processing techniques are described. Lastly, the most used sensor locations are highlighted together with the main application scenarios and experimental protocols tested for each of the three categories identified.

We decided to consider these three aspects (i.e., wearable system, signal processing, and application scenario) because there is a mutual influence among them. For instance, the processing technique must be chosen with respect to the sensor used because signals obtained with different sensors have different characteristics. In addition, different testing applications (e.g., at rest or during exercise) may require different processing techniques or the same technique (e.g., filter) but with different parameters; in particular cases, the application scenario may even require a specific type of sensor. Therefore, to give an overview of the context, we illustrate all three aspects, which must be carefully modulated with respect to each other in order to obtain a performant system for the intended application.

## 2. Precordial Vibrations Recording Using Accelerometers

### 2.1. SCG Signal

Different sensors may be used to record chest vibrations that originate from cardiac movements and pressure shocks. The most popular technique is based on accelerometers. These motion sensors should be tightly coupled to the subject’s chest wall surface to reliably detect its linear accelerations. Since 1960, the signal obtained by recording precordial vibrations from the chest wall surface using an accelerometer has been referred to as the SCG signal [15]. It traces the mechanical events occurring during the four main phases of a cardiac cycle. The SCG signal is recognized as the mechanical equivalent of the ECG signal. Although many studies during the past century have demonstrated that the morphology of the SCG signal changes due to different cardiac diseases, to date, the clinical acceptance of SCG is still hampered. The main obstacles to SCG clinical use are the instrumentation encumbrance, the lack of standardized sensor positioning, the influence of the inter- and intrasubject variability on SCG morphology, the lack of a standardized methodology to process the collected signal, and a reliable method for features extraction, useful to identify the underlying cardiac events [16,17]. However, recent research studies contributed to providing a deeper insight into these issues and proving the potential clinical feasibility of SCG for an early diagnosis of abnormal cardiac functions [18,19,20,21,22].

One of the main challenges in SCG studies is that SCG morphology appears to vary significantly, not only for cardiovascular pathologies but also due to other factors such as age, sensor locations, sex, and postural positions.

**Figure 1:**
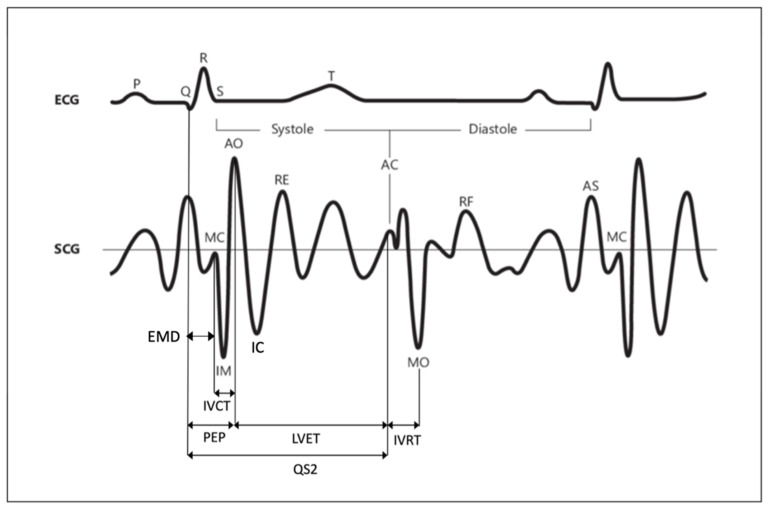
Typical SCG waveform and nomenclature with corresponding ECG signal. Adapted from [29]. Copyright 1993 with permission from Elsevier.

A typical SCG signal along with the corresponding ECG signal for two consecutive beats is shown in Figure 1. The SCG waveform shows clearly identifiable peaks that correspond to specific events in the mechanical activity of the heart, which are delayed in time with respect to the corresponding electrical events. The standard fiducial points of the SCG signal are aortic valve opening (AO), aortic valve closure (AC), mitral valve opening (MO), mitral valve closure (MC), and the IM point that occurs during the period of rapid change in ventricular pressure. Five additional features, namely, the rapid ventricular filling (RF), rapid ejection (RE) of blood from the ventricles, isovolumic contraction (IC), and peak of atrial systole (AS) can be eventually identified on the standard SCG waveform [23]. All the seismographic feature points highlighted in the literature are schematically reported in Table 1, followed by an explanation of the physiological event that they represent. Unfortunately, the annotation of SCG peaks and valleys is more challenging than ECG labeling. The field of electrocardiography has been in existence for over a century; hence, the technology used for ECG measurements has been widely assessed and innovated. In contrast, seismocardiography is a more recent technique still requiring improvements in the knowledge of SCG signal genesis and of the factors that affect its morphology (e.g., sensor locations, human motion, and respiration). Because of the SCG recent history, the literature is not yet consistent in terms of the location, definition, and annotation methodology of all the fiducial points of SCG signal. Several studies proposed SCG monitoring techniques for the evaluation of cardiovascular parameters (e.g., HR extraction and heartbeat duration), extraction of heart rate variability (HRV), and estimation of hemodynamic parameters. However, most of these works used the SCG signal in association with other clinically relevant techniques (e.g., ECG and PPG) for an early diagnosis of certain CVDs and detection of valvular defects (e.g., premature mitral valve closure, abnormal leaflet closure, and regurgitation of blood).

The reliable identification of SCG fiducial points enables an easy extraction of several cardiac time intervals (CTIs), including systolic time intervals (STIs). The STIs are as follows:

- The pre-ejection period (PEP), which is the time interval between electrical depolarization of the left ventricle (QRS on the ECG) and the onset of ventricular ejection;
- The left-ventricular period (LVET), defined as the time interval between the opening and closing of the aortic valve. It is the phase of systole during which the left ventricle ejects blood into the aorta;
- The QS2, which is the time period between the onset of the QRS complex and the first aortic vibration of the second heart sound. The sum of PEP and LVET gives the total time of electromechanical systole.

Both PEP and LVET are indices of myocardial contractility. In particular, PEP allows assessing how myocardial contractility is affected by the cardiac preload and afterload, while LVET is influenced by HR.

The extraction of these intervals from SCG has already been successfully demonstrated by comparing the results against an ECG signal used as a reference [24]. An accurate measurement of these intervals should be important since any deviation from the physiological pattern may correspond to an abnormality of cardiac functions. These time intervals should have a duration that varies within a range of normal values. For those intervals which are computed on the basis of a peak of the ECG signal, the normal delay can be interpreted as the physiological time difference between the electrical and mechanical aspects of cardiac activity. Even this electromechanical delay must vary within a range of normal values.

For instance, the LVET interval increases in patients with aortic valve dysfunction and decreases in patients with left-ventricular dysfunction. The PEP interval can increase as a consequence of left-ventricular failure, left-bundle branch block, or negative inotropic agents, and it can decrease as a consequence of aortic valve disease, low left-ventricular isovolumic pressure, or positive inotropic agents. The QS2 interval increases in patients with left-bundle branch block and aortic valve disease, and it decreases in the presence of positive inotropic agents [25]. 

In addition to STI, other clinically relevant time intervals that have been pointed out in the literature are as follows:

- The electromechanical delay (EMD), which is the interval between the ECG Q wave and the closure of mitral valve.
- The isovolumic relaxation time (IVRT), defined as the time interval between the end of aortic ejection and the beginning of ventricular filling.
- The isovolumic contraction time (IVCT), which is the interval between the closing of the atrioventricular valves and the opening of the semilunar valves.
- The pulse transit time (PTT), which is the time required for the travel of the blood pressure wave from one location to another. As PTT is inversely proportional to the blood pressure value, its evaluation is considered a promising method for continuous, noninvasive, and cuffless blood pressure monitoring. The most common type of PTT that can effectively estimate blood pressure is the time delay between a proximal-location pressure wave and a distal arterial-location pressure wave. This metric is called aortic PTT.

Even for IVRT, IVCT, and PTT, any deviation from the physiological duration may correspond to an abnormality in the cardiac mechanics. For instance, when the left-ventricular relaxation is defective, the decrease in left-ventricular pressure is slow, and this induces a delay in the normal crossover between the left-atrial and left-ventricular pressures. This phenomenon determines a delay in the opening of the mitral valve (i.e., MO fiducial point) and prolongs the IVRT. Thus, a prolonged IVRT is an early indicator of left-ventricular diastolic dysfunction. Moreover, as RE is a fiducial point that corresponds to arterial circulation, the SCG signal could also be exploited for stroke volume estimation, which is computed as the time integral of the flow rate in the systolic ejection period [26,27,28].

Some of these indices (i.e., PEP, EMD, and QS2), by definition, require the joint use of the mechanical signal (i.e., SCG) together with an optical or electrical signal (i.e., ECG and PPG) for their computation. In particular, the estimation of PEP and QS2 relies on the use of a reference ECG signal, while the estimation of the PTT interval relies on the use of a reference PPG signal. Only a few indices (i.e., LVET, IVRT, and IVCT) can be extracted from standalone SCG because they entirely rely on SCG fiducial points.

**Table 1:** Most common seismographic feature points that have been pointed out in the literature.

Fiducial Point	Physiological Event
**Aortic valve opening (AO)**	Aortic valve passively opens because of pressure differences on either side of the valve and allows the ejection of blood into the vascular tree
**Isovolumic contraction (IC)**	Event occurring in early systole during which the ventricles contract with no corresponding volume change
**Peak of rapid systolic ejection (RE)**	Rapid ejection of blood into the aorta and pulmonary arteries from the left and right ventricles, respectively
**Aortic valve closure (AC)**	Closure of the aortic valve at two-thirds of ejection
**Mitral valve opening (MO)**	Mitral valve opening when the left ventricle relaxes
**Peak of rapid diastolic filling (RF)**	The period in which the ventricle fills with blood from the left atrium from the onset of mitral valve opening to mitral valve closure
**Peak of atrial systole (AS)**	Peak of arterial blood pressure during systole, normally from 90 mmHg to 120 mmHg
**Mitral valve closure (MC)**	Mitral valve closure in correspondence with the left-ventricle contraction
**Isovolumic movement (IM)**	Ventricular isovolumetric contraction

### 2.2. SCG Signal Collection and Analysis

**Figure 2:**
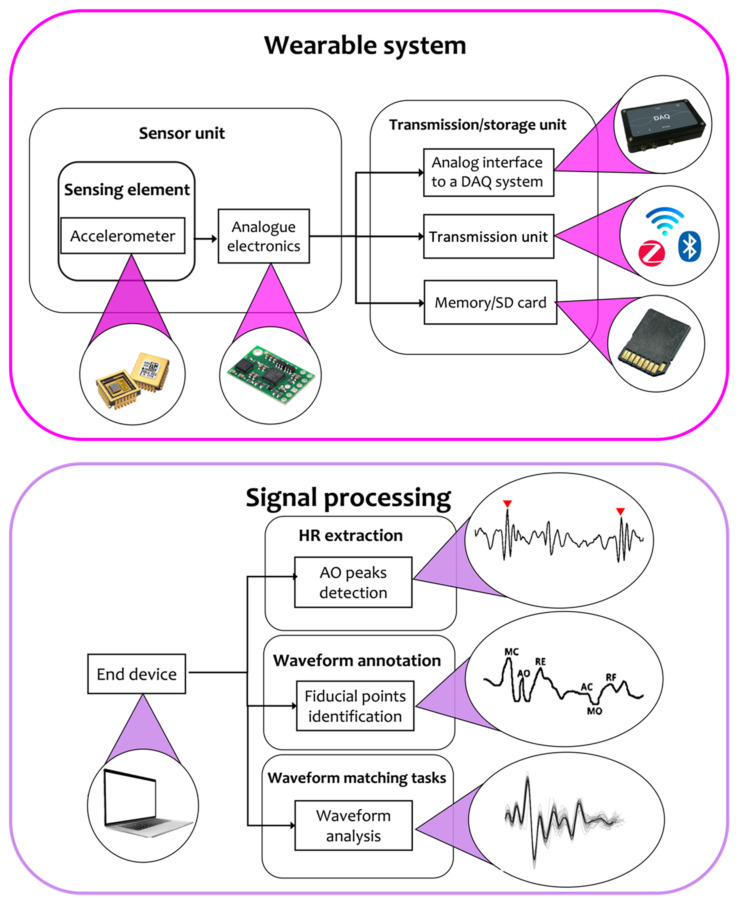
The two main blocks required to extract the information of interest: the wearable system with its building blocks (sensing element, analog electronics, and data transmission/storage unit) for signal collection, and signal processing for HR estimation and fiducial point extraction using accelerometers.

To extract the parameters of interest from raw data, two main blocks are required (see Figure 2): the wearable system with its own building blocks that include the sensor unit to record the SCG signal and the unit for data transmission and/or storage, and a signal processing block to process the signal and extract cardiac information (e.g., HR values and waveform fiducial points). Specifically, the output of the wearable system (i.e., the raw/semi-raw SCG signal) is given as an input to the signal processing block, and a processor/computer with a sufficient computational power is used to extract the valuable information from the SCG signal. In this section, further details about both these blocks are given together with a description of the most used sensor positions and experimental protocols proposed in the literature to assess the SCG-based wearable system performance in cardiac monitoring.

#### 2.2.1. Wearable Systems for SCG Monitoring

Accelerometers, along with gyroscopes, are inertial sensors that measure the acceleration of a proof mass, which leads to a change in some physical quantity depending on the nature of the sensing element (i.e., the capacitance between the proof mass and the substrate in capacitive accelerometers) [30]. Often, accelerometers are found in a triaxial configuration and measure linear accelerations along the three axes. The accelerometer can be modeled as a mass–spring–damper system; if a force is applied to the system, the outer shell accelerates while the internal mass (i.e., m) tends to remain stationary due to the principle of inertia and, therefore, the spring stretches. For details, see Section S1 of Supplementary Materials.

In the real world, no inertial accelerometer is composed of a mass and a spring, but it is an electromechanical device that converts mechanical motion into an electrical signal by means of a transduction element. According to the transduction element, accelerometers may be classified into three main categories: capacitive, piezoresistive, and piezoelectric [31,32]. In all three cases, as the small accelerometer mass reacts to motion, it places the transduction element into compression or tension.

Capacitance-based accelerometers are characterized by a simple design, insensitivity to temperature variations, and high sensitivity (i.e., maximum displacement of ~20 μm). They are ideal for measuring low-frequency motion where the g level is also low, such as vibrations. The sensing mechanism relies on a change in capacitance; a stationary plate, fixed to the housing, and a mobile plate, free to move inside the housing, form a capacitor whose value is a function of the distance between the plates. This change in capacitance is proportional to the acceleration that the inertial mass undergoes, and it is converted into an electrical output by a readout circuit [33,34].

Piezoelectric accelerometers exploit the piezoelectric effect of a quartz crystal; when an accelerative force is applied to it, the crystal produces a voltage that is proportional to the applied force [35,36,37].

Piezoresistive accelerometers are made of a piezoresistive material that gets deformed when a mechanical force is applied to it. In this case, the change in resistance is measured. These accelerometers are characterized by a high bandwidth, which allows them to measure high frequency, and a low sensitivity, which makes them unsuitable for vibration recording.

Today, accelerometers are miniaturized machines embedding both the mechanical and the electrical components for signal transduction (i.e., microelectromechanical systems, MEMS). The physical dimensions of MEMS can range from less than one micrometer to several millimeters [38]. For instance, piezoelectric MEMS accelerometers are made of lead zirconate titanate (PZT), a sensing element that produces a proportional electric charge under acceleration, allowing to measure vibrations.

According to the literature, SCG measurement systems can be divided into two main categories of sensing units: systems that use commercially available uniaxial or triaxial accelerometers, and systems that integrate these readymade accelerometers in custom circuitry elements for signal conditioning (e.g., analog filtering).

In recent studies, commercial one-axis ([39,40,41,42]) and three-axis ([22,43,44,45,46,47,48]) accelerometers with a weight ranging between 0.26 g and 43 g and a maximum operational level of 2000 g were used (the most used monoaxial sensor node is the high-sensitivity Brüel and Kjær model 4381 piezoelectric accelerometer [49], which has a weight of 43 g and a sensitivity of 10 pC/ms^−2^, while the most used triaxial accelerometer is the PCB Piezotronics sensor model 356A32 [50], which has a weight of 5.4 g and a sensitivity of 100 mV/g). 

In a few studies, custom systems for SCG recording were proposed [51,52,53]. In [51], a self-built system that includes three subsystems (i.e., SCG, ECG, and synchronous data collection subsystems) was designed. The multichannel SCG measurement subsystem is composed of multiple accelerometer sensing modules, whose core is the three-axis digital accelerometer LIS331DLH from STMicroelectronics [54], a microcontroller for data processing and conversion, which can communicate with multiple accelerometer sensing modules using an inter-IC (I2C) interface, digital-to-analog converters, and a synchronous data collection subsystem for data synchronization. In [52], a similar approach was adopted, featuring a custom printed circuit board (PCB) that includes a miniaturized MEMS accelerometer (ADXL335, ±3 g) for SCG recording and analog electronics for signal preprocessing (i.e., a preamplifier, a Butterworth low-pass filter with a cutoff frequency of 50 Hz, and a buffer). 

In all these studies, the collected data, once registered by the sensing element, are transmitted via the wireless module through a communication protocol (e.g., Bluetooth, Wi-Fi, or ZigBee) or via cable through a DAQ (e.g., iWorx 228 from iWorx Inc., Denver, NH, USA or NI-9205 from National Instruments, Austin, TX, USA) to a PC for signal processing and storage. In a few studies, the inertial sensors are provided with an analog interface for connection with the analog-to-digital converter (ADC) channel of a DAQ system that reads and converts the collected signals [40,41,44,48,51,55,56]. DAQ systems are also useful for a synchronized acquisition of signals from SCG sensor and reference instrument when used. As an alternative, the inertial unit is provided with a Bluetooth interface for data transmission. This case is quite common in custom realized PCBs. For instance, in [52], a wireless communication interface was integrated in the ad hoc designed PCB. In [45], acceleration and ECG signals were synchronized by means of a micro controller unit (MCU), which collects and streams data via TCP/IP over WiFi to a receiving client on a PC that stores the data for further offline processing. In [53], the sensor was connected to a wireless transmitter and a receiver to pick up seismocardiography and the pulse generated by the sensing circuitry using the ZigBee communication protocol. 

Rarely, raw data are stored on a separate device, memory, and/or SD card before transmission to the end-device used for data analysis, such as in [57], where a Freescale FRDM-KL25Z board was used to collect the data on a memory stick. 

#### 2.2.2. Signal Processing

The signal processing stage is generally dedicated to the identification of SCG fiducial points (already listed in Table 1) or to waveforms matching tasks. 

For what concerns the identification of SCG fiducial points, a few studies focused on the identification of the AO peak only (see Figure 1), which is the mechanical equivalent of the ECG R-peak and, thus, taken as a reference for the extraction of reliable HR/HRV values [30,33,45,48,51,53,57,58]. In recent years, for the estimation of the HRV index, the peak corresponding to the isovolumetric contraction (IC) has also been investigated. Further and more challenging analysis was aimed at the identification of other fiducial points in addition to AO and IC to achieve a reliable annotation of SCG waveform and an accurate estimation of CTIs [23,39,40,45,51,52,59,60]. 

Once the SCG trace has been recorded, a filtering stage is essential to better emphasize distinctive features and peaks on the raw signal for both the estimation of HR/HRV and the SCG waveform annotation or analysis.

For HR/HRV estimation purposes, Butterworth bandpass filters in the range 5–30 Hz [61], 6–25 Hz [48], or 4–50 Hz [57] or low-pass filters with a cutoff frequency of 35 Hz [47] and 40 Hz [53] were used to preserve the frequencies related to the heartbeat. Additional filters can be used to remove noise, respiratory contribution, and motion artefacts. In [47], for instance, a third-order Savitzky–Golay filter with a 100 ms time window was used to remove motion artefacts [62]. Referring to the SCG annotation, the implemented filters proposed in the literature are different from the previous ones. Most studies used a Savitzky–Golay, Butterworth, or finite impulse response (FIR) bandpass filter in the range 0.3–50 Hz [51], 0.3–40 Hz [40], 0.5–40 Hz [60], 0.8–25 Hz [63], 1–40 Hz [60], 1–35 Hz [55], 50–500 Hz [41], and 2–14 Hz [45]. A few works used a low-pass filter with a cutoff frequency of 30 Hz [42] or 90 Hz [41]. The most appropriate type of filter is generally chosen in accordance with the fiducial points to be extracted and according to whether they belong to the systolic or diastolic profile.

For HR/HRV estimation, SCG signals are usually compared with their concurrent ECG using windowing techniques and thresholding-based peak detection schemes to estimate AO peak location. However, these techniques may show a degradation in performance in the presence of noise, interferences, and inter- and intra-beat physiological variabilities. Moreover, all these methods require the identification of R peaks in the ECG signal to localize the subsequent AO peaks in the SCG signal. The addition of a reference instrument (i.e., ECG) leads to an increase in the system overall cost and a decrease in user comfortability. To address these issues, Choudhary et al. [64] proposed an SCG standalone algorithm for HRV analysis which entirely depends on AO peak detection. The algorithm exploits modified variational mode decomposition (MVMD) and a decision-rule-based postprocessing scheme to detect AO peaks and tachogram of AO–AO intervals to estimate HRV indices. The proposed method showed a strong correlation and agreement with HRV indices measured using the traditional ECG.

For what concerns HR monitoring, Gonzalez et al. made a comparison of the accuracy of different efficient heartbeat detectors derived from SCG signals collected from subjects in a quiet environment while the subject is lying still. The study found that the best detector, for its simplicity, is based on a narrowband bandpass filter [61].

For waveform annotation tasks and other types of waveform analysis, the most common techniques can be grouped into four main categories: temporal adaptive template-based [45,55], temporal envelope-based [39,40,52], machine learning (ML)-based [43,44,59,60], and visual inspection and comparison-based [41,42,51]. 

Because of the influence of subject anthropometric characteristics and sensor positioning on the SCG morphology, adaptive algorithms have been developed. These algorithms are mainly based on the extraction of a template from the ensemble of multiple cardiac cycles, as well as the application of search windows and temporal/amplitude thresholds to the extracted template for identifying fiducial points on SCG local extrema [45,55]. Fiducial points can also be identified using temporal envelope-based techniques in which the SCG envelope is used to locate peaks together with time and amplitude features for a slope within a search window of a few milliseconds. This operation can be performed with concurrent ECG/PPG or using SCG as a standalone solution [39,40,52]. For instance, in [39], multiple envelopes (i.e., HR, diastolic and systolic envelopes) were derived from the SCG signal at different stages of the algorithm using high-pass filtering and triple integration. The HR envelope was used to replace the ECG R-wave as a reference, the systolic envelope was used to locate AO, AC, and IM points, and the diastolic envelope was used to locate MO points. 

Another viable option for SCG waveform analysis is represented by ML techniques which can be exploited for a variety of different tasks from waveform annotation and comparison to SCG template generation or waveform-based classification tasks [43,44,59,60,65,66,67,68]. ML techniques are useful and mainly employed for waveform matching tasks, which may pave the way for automated diagnosis of HF based on the SCG morphology changes. For instance, in [59], Hsu et al. combined wavelet transform with deep learning models, ML classifiers, and distance metrics to perform SCG biometric matching tasks on 1 s long signals [59]. In this study, ECG was used to segment SCG into cardiac cycles and to annotate AO peaks. Baseline ML algorithms (KNN, SVM, decision tree, random forest, naïve Bayes, and AdaBoost) and deep learning architectures (AlexNet and ResNet-50) were trained with the training data as input and tested on the testing data to evaluate their performance in predicting the subject’s identity on the basis of a pattern matching approach. Performance evaluation was also performed using recognition rate and equal error rate for authentication issues. The best SCG biometric models were the bump wavelet-transformed pattern and the ResNet-50 model which had 0% equal error rate and 100% recognition rate. In the literature, ML approaches are usually applied to SCG signals collected from HF patients, due to their powerful potential to group similar signals into classes with common features and high similarities. For what concerns clinical studies that involved signal collection from HF patients, Inan et al. devised a method based on comparing the similarity of the structure of seismocardiogram signals before and after physical exercise using graph mining (graph similarity score). They found that the graph similarity score can assess HF patients’ state and it correlates to clinical improvements in 45 patients (13 decompensated and 32 compensated) [22]. This attempt demonstrated that wearable technologies for cardiac monitoring together with ML algorithms can assess compensated and decompensated HF states by analyzing cardiac response to submaximal exercise. In [68], the k-means algorithm was used to cluster SCG events associated with the mechanical processes corresponding to AS, MC, AO, RE, PE, AC, RF, IM, IC, and MO fiducial points using unsupervised ML techniques. Differently, in [65], the clustering capability of the k-means algorithm together with the waveform alignment capability of dynamic time warping (DTW) was used for SCG template generation. Here, SCG segments from both healthy and HF patients were clustered according to morphological similarities using the k-means clustering algorithm. Then, signal traces were aligned and averaged to build SCG waveform templates for the two groups using the DTW algorithm. DTW is a timeseries analysis method used to align signals and find similarities between signals. Therefore, this approach is often used to build SCG waveform templates. 

Visual inspection and comparison-based techniques try to identify SCG features using m-mode echo images or other signals as a reference or to correlate them with the physiological events identified in the corresponding reference signal/image. For instance, in [41], Sørensen et al. annotated fiducial points on the SCG signal and tried to correlate them with physiological events identified in ultrasound images. For all subjects, a mean SCG signal was calculated, and fiducial points (peaks and valleys) were manually annotated and labeled in the same way across all signals. These features were then correlated with eight physiological events from the ultrasound images. The results of this study showed that the physiological events do not always occur exactly at the fiducial points (i.e., local extrema of the SCG signal), but before or after. However, the main limitation in this study was the low temporal resolution of the ultrasound modalities compared to that of the SCG signal. Similarly, Lin et al. computed the timing of feature events as the time lag from both the ECG R peak in each cardiac cycle and the corresponding echo point [51]. In this study, a multichannel system was used to record the SCG signal from different measurement sites, underlying cardiac activities that are invisible to the conventional single-sensing modalities, such as left-ventricular lateral wall contraction peak velocity (LCV), septal wall contraction peak velocity (SCV), transaortic valvular peak flow (AF), transpulmonary peak flow (PF), transmitral atrial contraction peak flow (MFA), and transmitral ventricular relaxation peak flow (MFE). 

Although research is moving toward an automatic annotation of the SCG waveform, in most of the studies, the main limitation is the dependency to an additional signal (e.g., ECG, PPG, and ICG) not only to validate the proposed technique but also as a reference to identify certain fiducial points or to segment the signal into cardiac cycles. Only a few studies proposed a method that does not require any reference signal for SCG waveform annotation [11,40,69]. In [40], Khavar et al. proposed a delineation algorithm to detect IM, AO, and AC fiducial points with or without the electrocardiogram (ECG) R-wave as the reference point. The proposed algorithm generates HR, systolic, and diastolic envelopes using high-pass filtering, moving average filtering, extrema search window, and probabilistic decision making. However, in the SCG standalone case, a lower detection rate was observed. In [69], the authors proposed a standalone AO peak detection algorithm based on dominant multiscale kurtosis (DMK) and dominant multiscale central frequency (DMCF) in a multiresolution domain using wavelet. Exploiting DMK and DMCF-based criteria, probable AO peak-rich sub-bands are selected, and the signal is reconstructed. Then, the reconstructed signal is enhanced for AO peaks using weights, which are based on relative squared dominant multiscale kurtosis (RSDMK). Lastly, AO peaks are detected using a Gaussian derivative filtering-based scheme. In the validation stage, the proposed method showed a detection accuracy of 86% over approximately 4585 beats. 

From this analysis of the state of the art, it emerges that signal processing for SCG fiducial point identification is still facing three major limitations: the lack of a standard filtering technique in terms of the type of filter and range of frequency to be used, the lack of a reliable SCG standalone algorithm for an automated and reference-independent waveform annotation, and the lack of a consistent number of explorative studies on HF patients and real clinical settings. 

#### 2.2.3. Experimental Setup and Application Scenarios

This paragraph aims to describe the influencing factors that may have an impact on SCG waveform and the experimental setups that have been arranged to investigate the influence of these factors on SCG signal. These influencing factors mainly include human biological factors (e.g., age, gender, BMI, and somatic features), which determine the presence and consistency of the biological tissues that vibrations encounter during transmission, the subject’s posture and physical conditions (i.e., health status, physical fatigue) during signal acquisition, human motion and/or respiration, which are responsible for motion artefacts on SCG signal that may overshadow the rhythm signal and degrade signal quality, and sensor positioning, which may lead to different signal waveforms. Indeed, in the literature, SCG signals have been collected under various experimental conditions, reported here in terms of sensor placement and performed protocol. Consequently, signal processing approaches for the suppression of artefacts introduced by the influencing factors have been proposed.

**Figure 3:**
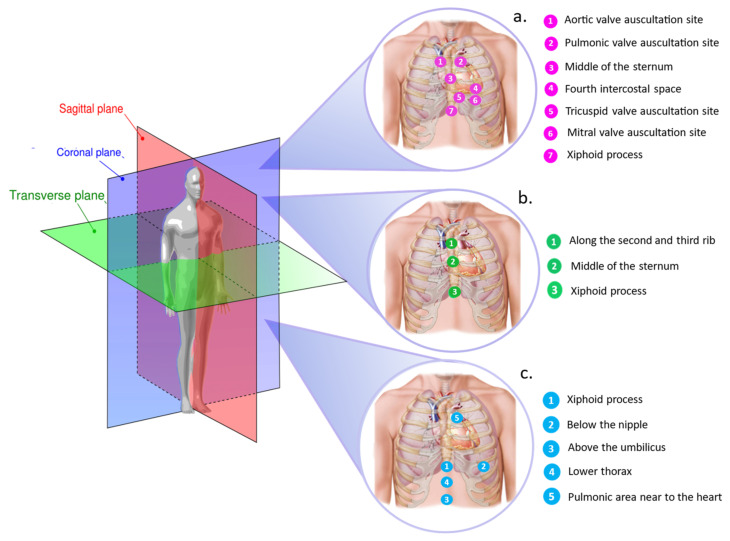
Distribution of accelerometer location sites for accelerometer-based (**a**), gyroscope-based (**b**), and FBG-based (**c**) wearable measurement in recent studies.

For what concerns sensor positioning, for precordial vibration monitoring, accelerometers are commonly attached to the patient’s chest at different placement locations on the sternum or in its proximity. The most investigated points include the sternum [45,47,66], the lower end of the sternum (i.e., xiphoid process) [41,46,52,55], the fourth intercostal space (IC4) near the left lower sternal border [43,44], and the four valvular auscultation sites (mitral, tricuspid, aortic, and pulmonary) [51,65]. Sensor placement locations that have been investigated in the literature are reported in Figure 3a, together with the measurement sites explored for the other sensor types (i.e., gyroscopes and FBGs) that we illustrate in more detail in the next sections. However, SCG recording is still lacking for standard measurement locations. Most of the studies proposed single sensing modalities consisting of an accelerometer placed on a single measurement site of the chest surface to collect the SCG signal from a single location on the chest. In a few cases, multiple accelerometers have been placed on specific landmarks of the chest to observe timing and pattern variations of feature points among the different recording sites [41,51].

Because the morphology of the SCG shows an inter- and intrasubject variability due to several factors (e.g., age, difference in the positioning of the sensor over the body, biological tissues crossed during wave propagation, and motion artefacts), most of the studies proceeded to collect data while the subject was in a resting state. Only a few studies included a period of physical activity in their experimental protocol [47,60,63]. However, signal processing was performed only on the pre- and post-exercise recordings, and physical activity was functional to create variations in the subjects’ HR and hemodynamics. For instance, Yang et al. proposed an experimental protocol that included climbing eight flights of stairs between two sessions of 5 min of resting [63]. However, SCG signals were collected during the two resting phases; hence, physical exercise was only used to create HR variations in the two sets of recordings (i.e., pre-exercise and post-exercise). In [60], Zia et al. asked the subjects to stand still for 5 min, to walk on a treadmill for 3 min at 3 mph, and to perform squat exercise for 90 s. Subsequently, the subject stood vertically and motionless for a 5 min recovery. Even in this case, useful data were limited to the recovery period because the subject’s hemodynamic state changed rapidly during this interval. 

Moreover, most of the studies were performed on healthy subjects in laboratory settings. Very few studies investigated the use of SCG signals to assess the clinical status of heart failure patients [22].

Several studies did not propose an experimental approach but focused on the development of innovative algorithms for data processing and adopted the records contained in publicly available databases to evaluate the performance of the proposed algorithm. For instance, in [46,58,59,64,69], the public CEBS database of PhysioNet containing the combined measurements of ECG, breathing, and SCG was used to test the performance of the proposed algorithm for HR estimation [70]. The details of the main studies that used accelerometers for recording precordial vibrations are schematically reported in Table 2.

## 3. Precordial Vibration Recording Using Gyroscopes

### 3.1. GCG Signal

Single-axis or triaxial gyroscope sensors (preferably MEMS) can be attached to the skin of the thorax and used to measure the three-dimensional angular velocities of the chest as a response to the motion of the heart.

**Figure 4:**
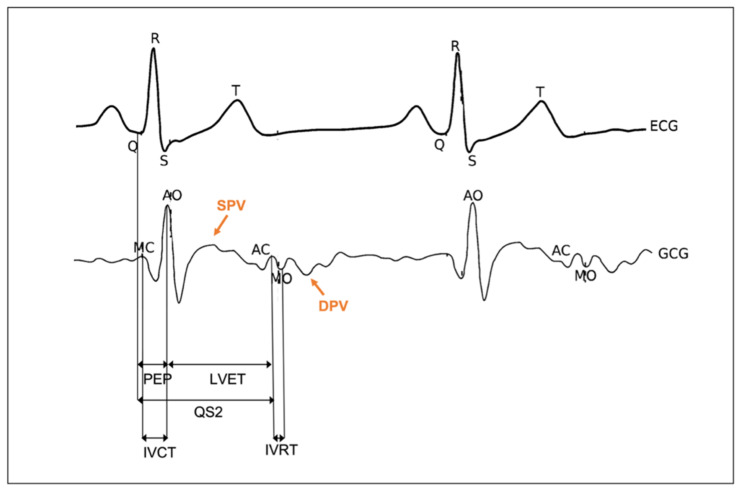
Typical GCG waveform and nomenclature with corresponding ECG signal.

The signal obtained by recording precordial vibrations from the chest wall using a gyroscope is commonly known as the GCG, which indicates the rotational movement of the chest in response to the cardiac activity (see Figure 4). The GCG is commonly collected together with SCG and/or BCG to constitute so-called mechanocardiography (MCG) or vibrational cardiography (VCG). Hence, although SCG and GCG result from the same physiological source, they represent different aspects of such analysis. GCG and SCG can be treated as complementary techniques, and the use of a gyroscope sensor can help improve the automated interpretation of SCG signals to estimate HRV, CTIs, and annotation of waveforms. 

The GCG is a low-frequency mechanical signal classified as a local pulse signal measured in degrees per second (i.e., °/s) [71]. GCG has a frequency range of 1–20 Hz and amplitude range within a few dps. GCG signal can be either registered on one or three axes of rotation (i.e., horizontal or *x*-axis, vertical or *y*-axis, and dorsoventral or *z*-axis), and each axis has a distinctive signal pattern. The *x*- and *y*-axes of the GCG have a similar waveform shape among different subjects and measurement devices. The GCG signal is generally less sensitive than the SCG signal to inter- and intrasubject variability [72]. Usually, the *y*-axis GCG signal has the highest signal-to-noise (SNR) ratio and, thus, it is taken as a reference for gyrocardiography. 

Since the GCG and SCG waveforms result from the same physiological events, traceable to cardiac mechanical processes, the peaks and valleys of the GCG signal reflect the same physiological events of SCG waves in the cardiac cycle, including mitral valve opening (MO) and closure (MC), isovolumetric contraction (IC), rapid ejection (RE), aortic valve opening (AO), and closure (AC). 

On the basis of these fiducial points, the isovolumetric contraction time (IVCT), isovolumetric relaxation time (IVRT), STI (i.e., PEP, LVET, and QS2), and other relevant indices of cardiac contractility and hemodynamics (e.g., stroke volume and cardiac output) can be estimated. In particular, LVET can be measured as the time interval between AO and AC fiducial points, and the PEP interval can be determined by calculating the time between the ECG Q-wave and AO. 

As GCG is a velocity signal, its waveform also reflects systolic peak velocity (SPV) and diastolic peak velocity (DPV), which can be annotated taking as a reference systolic myocardial velocity (Sa) and early diastolic velocity (Ea) indices in tissue Doppler imaging (TDI), which in turn are a measure of longitudinal systolic and diastolic function. Accurate estimation of the timings of tissue velocities can be clinically important, as it enables, for instance, the computation of myocardial dispersion, which reflects the heterogeneity of myocardial systolic contraction and can be used as an indicator of exposure to arrhythmias in different heart diseases (e.g., heart failure, ischemia, and infarction). Tadi et al. demonstrated the utility of assessing the timing agreement between the integral of the GCG *y*-axis signal (i.e., angular displacement) and 3D speckle tracking strain measurements, since strain is a function of position, which is in turn the integral of velocity. Thus, maximal angular displacement points may be useful for estimating the myocardial mechanical dispersion [72].

In the past few years, many studies focused on correlating GCG peak timings with the timings of peak tissue velocities; however, future research studies may be focused on investigating information that can be extracted from the magnitude of the GCG signal.

### 3.2. GCG Signal Collection and Analysis 

**Figure 5:**
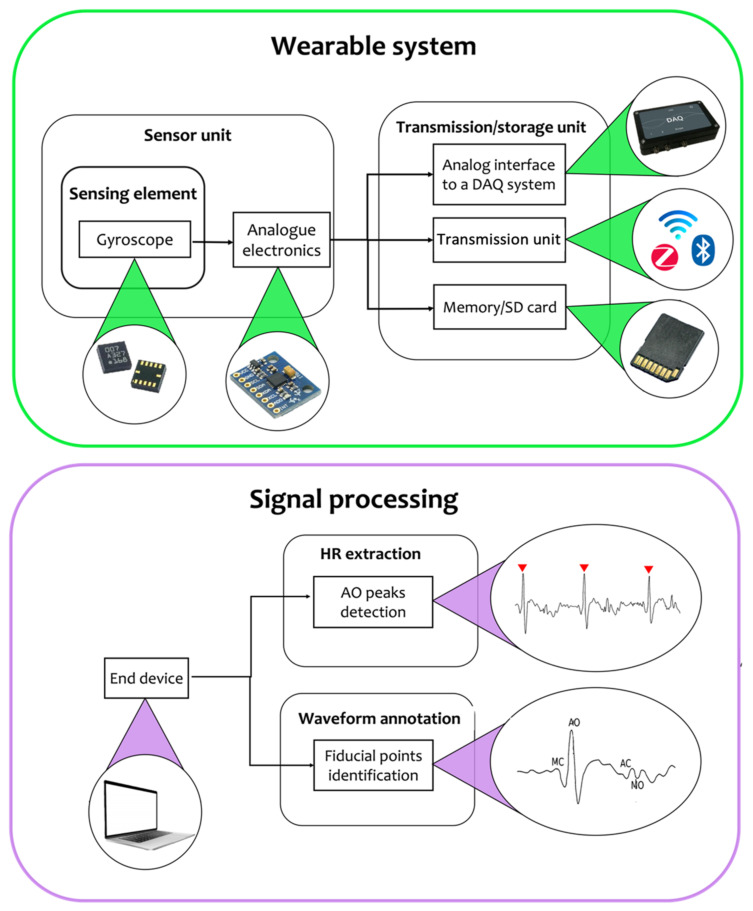
The two main blocks required to extract the information of interest: the wearable system with its building blocks (sensing element, analog electronics, and data transmission/storage unit) for signal collection and signal processing for HR estimation and fiducial point extraction using gyroscopes.

Like accelerometer sensors (see Section 2.2), even for gyroscope sensors, two main blocks are required to extract the parameters/time intervals of interest from raw signals (see Figure 5): the wearable system and the signal processing block. As gyroscopes are electronic inertial sensors, the building units that compose the wearable system are the same as those used for accelerometer-based wearables: the sensor unit with optional analog electronics and the storage and/or transmission units, used to transfer data to the end device employed for signal processing.

In the next sections, we describe the working principle of gyroscopes, and we give details about the most constitutive elements of the wearable systems proposed for GCG recording. Then, we highlight the main signal processing state-of-the-art techniques, the application scenarios (with particular regard to the performed experimental protocols), and the main influencing factors.

#### 3.2.1. Wearable Systems for GCG Monitoring

A gyroscope is a sensor of angular motion able to measure its own angular velocity or rate of gyration around a particular axis. It is often installed on a moving object to record its angular velocity. Traditional mechanical gyroscopes are composed of a toroid-shaped rotor that rotates around its axis, remaining parallel to itself and opposing any attempt to change its orientation. Today, the most used gyroscope is the MEMS gyroscope, which is characterized by miniaturized dimensions, low cost, low power consumption, and high accuracy. 

MEMS gyroscopes can be modeled as mass–spring–damper systems with a mass that moves along two orthogonal mechanical excitation modes (see Supplementary Materials Section S2). The in-plane rotation of a rigid body in a three-dimensional space can be described using Euler angles (*φ*′, *ϑ*′, and *ψ*′). Angular velocities $\omega_{x},\;\omega_{y}$, and $\omega_{z}$ generated by rotation along the *x*-, *y*-, and *z*-axes are related to Euler angles as follows:

(1) $$\left[\begin{array}{c} \varphi^{\prime }\\ \vartheta^{\prime }\\ \psi^{\prime } \end{array}\right]=\left[\begin{array}{c} 1\\ 0\\ 0 \end{array}\;\begin{array}{c} \sin\varphi \tan\vartheta \\ \cos\varphi \\ \sin\varphi \cos\vartheta \end{array}\;\begin{array}{c} \cos\varphi \tan\vartheta \\ -\sin\varphi \\ \cos\varphi \cos\vartheta \end{array}\right]\left[\begin{array}{c} \omega_{x}\\ \omega_{y}\\ \omega_{z} \end{array}\right].$$

Precordial vibrations cause very small angular velocities of deviation; thus, the gyroscope used for this application should be highly sensitive and should respond to very small variations in angular velocities [73].

Other parameters that characterize gyroscope sensors are the following:

- Angle random walk (ARW), which describes the error resulting from broadband white noise, which is caused in MEMS devices by detection electronics.
- Bias offset error, which is the nonzero output of the gyroscope when the input rotation is null. This static error is typically 25 °C for an ideal environment, and it can be easily corrected.
- Bias instability, which is the instability of the bias offset at constant temperature and in an ideal environment. It introduces a dynamic error difficult to compensate for, and it strongly affects sensor accuracy over a long time.
- Temperature sensitivity, which defines performance changes over temperature changes.
- Shock and vibration sensitivity, which denotes the degradation in performance caused by vibration and shock inputs.

The sensor metrological characteristics required depend on the specific application and operating conditions. However, bias drift is the most limiting factor for all kinds of gyroscopes [74]. 

In the GCG recording application, the gyroscope is mechanically coupled to the subject’s chest. Cardiac vibrations, which are projected outward along the dorsoventral axis, rotate the sensor in the two spatially coupled gyration axes. Up to 60% of cardiac vibrational energy is contained in the gyration signal, resulting in a higher noise rejection ratio than acceleration data. 

As GCG is an emerging technique, gyroscope sensors were only used in a few studies, and they were usually employed to complement information obtained from SCG signals [11,60,63,75]. D’Mello et al.’s work validated the benefit of the SCG–GCG combined use (i.e., VCG). This approach leverages the mutually orthogonal information that can be obtained from all six degrees of freedom, enabling a comprehensive analysis of cardiac vibrations [11]. 

For this purpose, the use of inertial measurement units (IMUs), embedding both a gyroscope and an accelerometer together with a magnetometer, is recommended. For instance, in [63], an IMU consisting of a three-axis MEMS accelerometer, a three-axis MEMS gyroscope, and a magnetometer was used for GCG recordings to enable a complete nine-DoF solution. The overall weight (23.6 g) and dimensions (51 mm × 34 mm × 14 mm) of the IMU are comparable to those of a single sensor node. Raw or pre-processed data from IMUs can be transferred to an end device for signal postprocessing via wireless or wired connection. For instance, in [63], all IMU and ECG measurements were simultaneously recorded and wirelessly transmitted via Bluetooth to a laptop, while, in [8], data were transmitted serially to a computer via USB cable using an Arduino Leonardo microcontroller. Otherwise, data can be stored locally using an onboard memory such as in [75], where all measurements from a six-degree-of-freedom IMU were stored on a memory card and processed later using custom-made software.

#### 3.2.2. Signal Processing

Gyrocardiography is a newborn technique for precordial vibration monitoring; therefore, most studies explored the feasibility of the identification of standard peaks on repeating patterns to find correspondence with SCG fiducial points and with the underlying physiological events [63,72,76]. A few studies also investigated the accuracy and reproducibility of CTI estimation from the proposed GCG fiducial points. For instance, Dehkordi et al. delineated, on the GCG *x*- and *y*-axis waveforms, five fiducial points associated with the opening and closure of the aortic and mitral valves and compared the manually annotated points with the corresponding events on TDIs [76]. Then, the CTIs and Tei index were calculated on the basis of the found fiducial points and compared to the TDI-based reference timings for validation. GCG *y*-axis points appeared to provide better estimates for CTIs than GCG *x*-axis points. Similarly, Tadi et al. attempted the identification of fiducial points and the estimation of CTIs (i.e., IVCT, IVRT, PEP, LVET, and QS2) on the *x*- and *y*-axis GCG signals, which are typically of better quality [72]. These studies demonstrated that triaxial GCG provides reliable fiducial points for cardiac events and reliable measurements of CTIs. A few recent studies took advantage of these waveform labeling attempts to extract the HR and HRV indices [11,77,78]. 

As for SCG signals, in this case, a filtering stage is fundamental prior to the actual signal processing and analysis, to remove bias, underlying trends, and high-frequency noise. However, despite the filtering stage, a significant amount of noise, related to the in-band noise, will remain. 

For waveform annotation tasks, Butterworth bandpass filters with 0.5–20 Hz [75], 0.8–25 Hz [63], and 1–20 Hz [72] frequencies were used. For HR/HRV estimation, in [77], a third-order Butterworth bandpass filter with a bandpass of 4–50 Hz was used to filter the raw GCG signals; then, a moving average FIR filter with the window width of 15 ms was used to smooth the filtered signals, and a third-order Butterworth bandpass filter with cutoff frequencies of 1 Hz and 40 Hz was applied to the beat detection results. In alternative, D’Mello et al. used a high-pass brick wall filter with a cutoff frequency of 0.4 Hz to filter the raw GCG signals for HR analysis [11]. 

Currently, gyrocardiograms are used to supplement the seismocardiogram signal rather than replacing it in HR estimation, feature extraction, and time interval estimation tasks. Indeed, GCG signal processing was primarily addressed to the identification of fiducial points for waveform annotation and CTI estimation [11,63,78,79]. In this process, it was necessary to use SCG or other signals (e.g., impedance cardiogram, ICG) as a reference to define a correct labeling of the GCG signal.

In [63], GCG signal and its first derivative (denoted as DGCG) were considered for both comparison and similarity analyses with SCG. Using impedance cardiography (ICG) and ECG as reference signals, a method for the identification and annotation of GCG and DGCG in both *x*- and *y*-directions was designed. 

First, 14 time differences between GCG/DGCG and SCG peaks were computed using pre- and post-exercise recordings. Results of the comparison analysis showed that the correlation between the annotated fiducial points (i.e., IM, AO, and AC) and the reference points is relatively high. Moreover, there were no significant differences between pre-exercise and post-exercise measurements regarding the identification of these fiducial points, which suggests that the identification is stable under mild heartrate variations. In addition, it was found that the DGCG_Y provides the best estimation of LVET and PEP.

Then, GCG and DGCG recordings were compared for similarity with SCG in both the time (i.e., correlation coefficient) and the frequency (i.e., frequency response assurance criterion, FRAC) domains using the most common measures in the area of vibration analysis. 

Results of the similarity analysis showed that taking the first derivative of GCG_X increases the similarity with SCG in both the time and the frequency domains. This means that, when a gyroscope is used to record precordial vibrations, the output signal can be derived one time to obtain a signal that is more similar to the standard SCG and its fiducial points.

Another viable option for signal processing consists of accelerometer and gyroscope signal fusion. For instance, in [75], a standalone (i.e., ECG-independent) technique for heartbeat detection that benefits from fusing SCG and GCG signals was proposed. The algorithm removes motion artefacts, selects the best axis from multiaxial accelerometric and gyroscopic signals, detects the location of beats using two detection techniques based on the signal envelope and morphological characteristics for both signal types, and finally merges the detected beat locations using both SCG and GCG signals to obtain the final estimate of beat positions. The algorithm removes artefacts by dividing each signal into 10 s epochs, computing the FFT for each epoch, and smoothing the signal with a moving average filter of 10 samples. The beat detection method includes two sub-algorithms, namely, wavelet enhancement and clustering. The algorithm performance was tested on both healthy subjects and patients with heart disease, and the average sensitivity and precision of the beat detection were 99.9% and 99.6% for the healthy subjects and 96.1% and 95.6% for the heart disease patients, respectively. In this study, the ECG signal was used for the validation task only and the average root-mean-square error (RMSE) between the mechanical and ECG inter-beat intervals was 5.6 ms for the healthy patients and slightly higher for the heart disease patients. 

From this analysis of the literature, it emerges that GCG signal processing for fiducial point identification is still facing the following limitations: the lack of a standard filtering technique in terms of type of filter and range of frequencies, and the lack of a standard sensor positioning protocol. However, early studies on automated methods for a human and reference-independent GCG annotation demonstrated the feasibility of HR extraction from standalone SCG/GCG and the validity of sensor fusion/combination to enhance the performance in heartbeat detection. 

#### 3.2.3. Application Scenarios and Influencing Factors

As for the SCG signal, the most investigated influencing factor is the sensor positioning. Gyroscope-based wearable systems in the literature have predominantly been attached to the patient’s chest in correspondence of three anatomical landmarks: the xiphoid process [11], the middle of the sternum [60,72,75], and along the second and third rib on the sternum [63]. Sensor locations investigated in the literature for GCG monitoring are reported in Figure 3b.

Although, in the literature, there are limited examples of experimental trials involving the use of a gyroscope, signal acquisition has been explored under various experimental conditions. For instance, data collection has been performed both with the subject in a resting position [72] and before and after a few minutes of physical activity [63]. Moreover, Kaisti et al. decided to thoroughly evaluate the performance of the method they proposed for standalone heartbeat detection using mechanocardiograms, not only on signals collected from healthy subjects but also on more challenging signals recorded from heart disease patients in a clinical environment. The promising results obtained in this study [75] suggest further analysis on signals acquired in a clinical scenario.

Several studies did not build up an experimental protocol for data acquisition but exploited readymade GCG signals available in public databases. For instance, Siecinski et al. performed data analysis on the “Mechanocardiograms with ECG Reference” dataset by Kaisti et al. [75] publicly available from the IEEE DataPort data repository [80]. The dataset contains 29 simultaneously recorded ECG, SCG, and GCG signals collected from 29 healthy male volunteers with the following demographic data (expressed as minimum, maximum, mean, and standard deviation): age (23–41, 29.5 years), height (170–190, 179, 5 cm), weight (60–98, 76.11 kg), and BMI (18–30, 24.3 kg/m^2^). The details of the main studies that used gyroscopes or IMU platforms for recording precordial vibrations are schematically reported in Table 3.

## 4. Precordial Vibrations Recording Using Fiber Bragg Grating Sensors

### 4.1. Strain-Derived SCG Signal

To overcome the limitations of traditional MEMS sensors in this application (e.g., stiffness, encumbrance, nonoptimal skin adherence, and compliance with the body surface), the use of flexible sensors integrating different types of sensing elements into soft matrices (e.g., conductive textiles and fiber optic sensors) has been proposed [81,82,83,84]. Usually, substrates are thin plastic films made of polyester (PET), polyimide (PI), polyetherimide (PEI), parylene, polyethylene naphthalate (PEN), or intrinsically stretchable elastomers such as polydimethylsiloxane (PDMS), Ecoflex, Dragon Skin, thermoplastic urethane (TPU), and styrene–ethylene–butylene–styrene (SEBS). These materials have excellent mechanical properties under bending, folding, or crumpling. For instance, You et al. fabricated a stretchable electronic skin made of Au nanoparticles and elastomer PDMS to sense the deformations of the chest wall caused by the vibrations induced on its surface by the heart apex [85]. However, most of these systems are not able to sense microstrains induced on the chest surface by the heart beating. Therefore, there are a few studies which focused on flexible wearable sensors for cardiac monitoring via strain sensing. The most popular ones are those based on fiber Bragg gratings (FBGs), due to their intrinsic advantageous properties, such as high sensitivity, frequency response, biocompatibility, chemical inertia, and immunity to electromagnetic interferences, which make them usable even in harsh environments (e.g., under high-pressure, high-temperature, and strong-magnetic-field conditions).

In cardiac monitoring applications, the output of FBG sensors (i.e., the Bragg wavelength—λ_*B*_) shifts accordingly with precordial vibrations. The FBG encapsulation into flexible configurations enables an optimal mechanical coupling between the sensor and the skin, guarantying a better transduction of chest wall deformations into Bragg wavelength shifts (Δλ_*B*_). These fiber optic sensors are intrinsically sensitive to strain (ε); thus, they capture cardiac vibrations in the form of strain measurements induced by chest deformations in response to the mechanical events of the cardiovascular system. State-of-the-art studies refer to this signal as SCG even if strain-derived.

### 4.2. Strain-Derived SCG Signal Collection and Analysis

**Figure 6:**
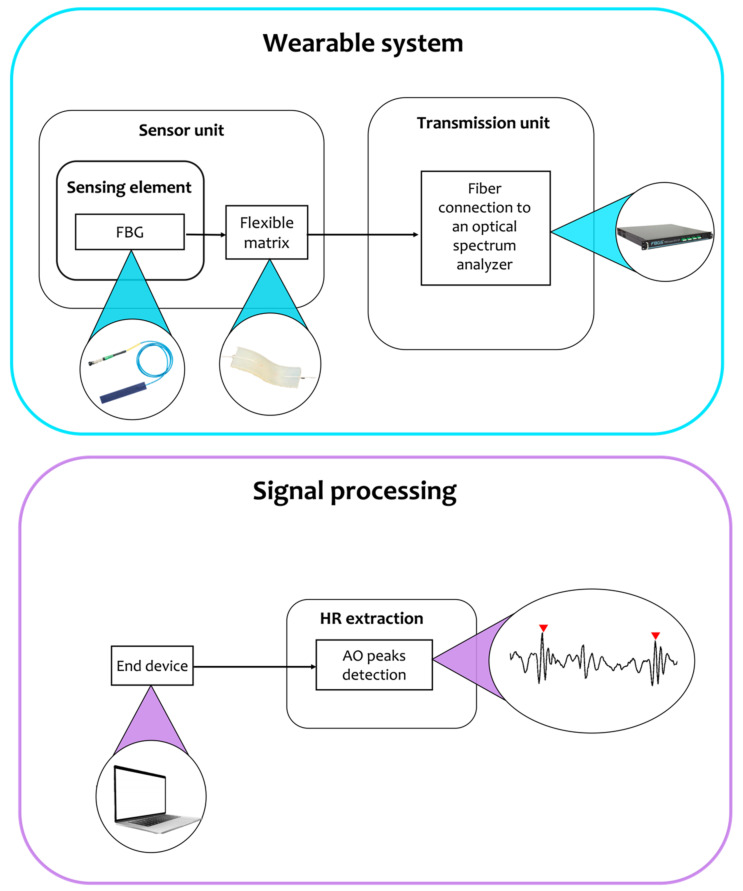
The two main blocks required to extract the information of interest: the wearable system with its building blocks (sensing element and data transmission unit) for signal collection, and signal processing for HR estimation and fiducial points extraction using FBGs.

As in previously described cases, even SCG monitoring based on FBG sensors relies on two main blocks (see Figure 6): a wearable system and a signal processing block for HR extraction, which is the principal aim of FBG-based studies present in the literature.

The FBGs are the widespread fiber optic technology employed for monitoring precordial vibrations. They are preferred to traditional electrical and mechanical sensors due to their advantageous metrological properties [86] and immunity to electromagnetic interferences, which allows their use in harsh environments such as MRI procedures in the clinical application [87]. For what concerns cardiac monitoring, the high *ε* sensitivity (up to 1 pm/με) and linearity in a wide range of *ε* values, short response time (<10 ms), and proper frequency response of FBGs make them good candidate for detecting the small and rapid heart-induced deformations of the chest surface. Other advantages in the use of FBG sensors for cardiac monitoring via wearable systems are related to their miniaturized size, ultralightweight, chemical inertness, capability to work in harsh environments, long-term stability, and durability (i.e., the average lifespan of etched gratings is 30 years). Usually, for SCG monitoring, FBGs are directly glued on smart textiles and, recently, encapsulated into polymeric matrices to obtain more robust systems. The sensor positioning, as well as the matrix shape and dimensions, can be carefully designed to optimize the FBG response to *ε*. 

The main building blocks for SCG monitoring using FBGs are the wearable system for SCG acquisition from the subject and the signal processing stage for AO peak detection and HR estimation. FBG-based wearables, in the most general case, are composed of three main building blocks: the sensor, which is the grating inscribed into the core of the optical fiber, the coating matrix, which confers the properties of flexibility, stretchability, and robustness to the sensor, and the optical spectrum interrogator, which manages both the processes of fiber light excitation and the examination of the reflected spectrum. The post-acquisition stage with the use of a dedicated software then leads to the results of the analysis performed, which is limited to HR estimation in the current state of the art. In this paragraph, we describe the working principle of FBGs, and we give details about the studies in the literature that exploit these sensors for cardiac monitoring, in terms of wearable systems, signal processing and application scenarios, inclusive of the possible influencing factors.

#### 4.2.1. Wearable Systems for Strain-Derived SCG Monitoring Using FBGs 

Basically, an FBG sensor is an optical strain gauge and can be considered as a short segment of an optical fiber (usually 3–6 mm or smaller). When interrogated with a broadband spectrum of light, it reflects a narrow spectrum centered around a specific wavelength (i.e., the Bragg wavelength, λ_*B*_) and the resting part of the light traveling along the fiber is transmitted. The back-reflected spectrum is centered around the so-called Bragg wavelength, $\lambda_{B}$, which depends on the effective refractive index of the fiber core and on the grating spatial period, Λ.

(2) $$\lambda_{B}=2\Lambda \;\eta_{eff}.$$

Both the terms in the Bragg condition are sensitive to *ε* and temperature (T); thus, the use of a proper configuration and design allows the estimation of these parameters by monitoring changes in λ_*B*_ (see Supplementary Materials Section S3). Indeed, when an FBG is exposed to *ε* and *T* changes, a variation in Λ and *η_eff_* occurs causing a shift of λ_*B*_ (Δλ_*B*_).

(3) $${\Delta \lambda }_{B}={\Delta \lambda }_{B}{}^{mech}+{\Delta \lambda }_{B}{}^{therm}.$$

The first term in Equation (3) represents the *ε* effect on an optical fiber (${\Delta \lambda }_{B}{}^{mech}$), and the second term represents the effect of *T* (${\Delta \lambda }_{B}{}^{therm}$). These terms can be expressed as

(4) $${\Delta \lambda }_{B}{}^{therm}=\lambda_{B}\;S_{T}\;\Delta T,$$

(5) $${\Delta \lambda }_{B}{}^{mech}=\lambda_{B}\;S_{\varepsilon }\;\varepsilon ,$$

where $S_{T}$ is the sensitivity to *T* changes, and $S_{\varepsilon }$ is the sensitivity to *ε*, which are determined by means of a calibration process. Hence, FBG sensors are intrinsically sensitive to both these parameters, but several strain–temperature discrimination techniques and particular sensor encapsulation packages have enabled the development of sensors that are selectively sensitive to only one of the two physical quantities [88,89]. For details see Section S3 of Supplementary Materials.

During cardiac monitoring applications, the FBG should be highly sensitive to *ε*, and the influence of *T* should be considered negligible [90,91].

The sensor response to *ε* is encoded in a spectral magnitude (i.e., $\lambda_{B}$); thus, the FBG response is absolute and self-referential, and it does not depend on environmental noise or power fluctuations in the light source. By interrogating the Bragg wavelength λ_*B*_, the physical quantity that acts as an input perturbation (e.g., strain) can be quantified.

In this application, the single-parameter sensor is adherent to the subject’s chest, and the FBG’s output (i.e., Δλ_*B*_), which changes in response to the mechanical strain applied to the sensor grating by precordial vibrations, can be displayed over time providing a similar SCG signal. 

Usually, FBGs are directly attached to smart textiles (e.g., T-shirts and bands) encapsulated into polymeric PDMS [92], polymethyl methacrylate (PMMA) [88], Dragon SkinTM 20 silicon rubber [93,94], or more recently, into 3D printed materials [95]. These coating matrices may have different shapes easily conferred by 3D molding injectable techniques or 3D printing, from simple rectangular to the dogbone shapes. Each design, as well as the FBG positioning into the matrix, can be customizable to emphasize the *ε* transmission to the encapsulated grating. For instance, in [96], a custom cone-shaped structure made up of polyvinyl chloride (PVC) was developed and attached to the moving end of a micrometer and to a flexible diaphragm to reach the chest muscle with the minimum discomfort for the subject. Not only single sensing modalities but also multisensor systems based on FBG arrays have been proposed. These novel solutions exploit the multiplexing capability of FBG sensors that allow having multiple sensing elements distributed along the same optical fiber in an array configuration to perform quasi distributed sensing. The possibility to customize the sensor by means of an ad hoc manufacturing process gives great freedom in terms of dimensions, materials, and shapes, which allows adapting the sensor to the needs of the specific application. When the FBG is embedded into the flexible coating, the elastic and thermal properties of the material influence the sensor sensitivity to temperature and strain; thus, a static and dynamic characterization process is needed to determine the novel properties of the ad hoc fabricated sensor. Chest deformations do not directly impact the silica fiber, but they stretch the surface of the 3D printed polymer, which transmits the stretching effect to the silica fiber leading to a change in the FBG sensor output (∆λ_*B*_).

#### 4.2.2. Signal Processing

In the literature, FBG sensors for SCG monitoring have been developed and used to estimate HR by measuring the *ε* signal that results from the displacement (i.e., from 0.2 mm to 0.5 mm) occurring on the subject’s chest during heart beating [5]. These displacements are lower than those caused by the breathing activity. Hence, a filtering stage is required prior to the actual signal processing and HR analysis to remove the respiratory contribution and reveal the heartbeat signal. The filters used in the literature are bandpass filters with lower cutoff frequencies [86] of 0.8 Hz, 3 Hz, and 5 Hz and higher cutoff frequencies of 2 Hz, 20 Hz, and 30 Hz [88,93,94,95]. Often, upper and lower envelopes are additionally performed on the filtered signals to better emphasize the AO-related peak on the SCG signal [86]. The filtered signals are processed by following two main approaches: time- and frequency-domain analysis. In the first case, a typical approach consists of detecting the local maxima in the processed signal and then computing HR as 60/Tc (where Tc is the time elapsing between two consecutive maximum points). In the latter case, HR is calculated in the frequency domain by performing a spectrum analysis based on fast Fourier transform (FFT) or Welch’s method. HR is estimated from the dominant frequency of the power spectrum.

From this analysis of the literature, it emerges that SCG signal processing and analysis using FBG sensors is still lacking standardized filtering techniques, postprocessing analysis, and sensor positioning guidelines. Moreover, the proposed studies estimated only HR, while no waveform analysis was performed to identify fiducial points on the collected signal.

#### 4.2.3. Application Scenarios and Influencing Factors

FBG-based sensors are tightly fixed to the subject’s chest in correspondence with the chosen measurement point using adhesive tape or a contact strip such as a Velcro fasten belt, allowing the mechanical stress to be transferred to the sensing element. In the literature, different measurement points on the frontal plane have been investigated (see Figure 3c): xiphoid process [94,95], the area of the sternum below the nipple [94], the area above the umbilicus [86,94], lower thorax [93,96], and pulmonic area near to the heart [88]. As for SCG recording with accelerometers, FBG-based SCG monitoring is still lacking for standard measurement locations and filtering stages. Moreover, the majority of the studies focused on single sensing modalities consisting of a single FBG element, bare or embedded into polymer matrices, to collect the SCG signal from a single measurement site on the chest surface. Recently, Lo Presti et al. proposed the use of a multi-sensor system consisting of an array of four FBGs and investigated the influence of sensor positioning on the SCG recording. In particular, they placed the array on three different positions to obtain simultaneous SCG recordings from 12 total measurement sites and observe waveform variations among the different recording sites [86]. In this study, the performance of the wearable system was evaluated through a comparative analysis between the single-sensor and the multi-sensor approach, and results showed that the signal obtained by averaging the signals collected from all the FBGs provided a better performance in HR estimation than each FBG sensor considered individually. These results demonstrated that a multi-sensor approach obtained by summing the outputs of multiple sensors can improve the system performance in HR estimation.

Until now, the feasibility of noninvasive FBG-based SCG monitoring has been demonstrated on stationary subjects only and mainly during apnea stages to automatically discharge the respiratory contribution on the FBG raw data. In general, if signal acquisition is performed during normal breathing, the sensor is strained by chest wall deformations induced by both respiratory and cardiac activities; therefore, a more challenging signal processing is required to exclude the respiratory effects [96]. Otherwise, if the subject is asked to hold their breath during the acquisition session, only the cardiac contribution is registered. 

Together with the multiplexing capability and the high metrological properties, another interesting feature of FBGs is their immunity to electromagnetic interferences that enables the cardiac monitoring even in harsh environments (e.g., during magnetic resonance imaging (MRI) procedures). For instance, Nedoma et al. carried out experimental tests of SCG monitoring in MRI environment and found that the metal-free design of FBG sensors does not pose any threat to the patients undergoing MRI exam and has no influence over the quality of imaging [88]. Moreover, the FBG output signal is identical to that ne obtained without any electromagnetic interference; thus, the measured signal is not affected by the 1.5 T magnetic field. Although the use of FBG sensors for cardiac monitoring is at the beginning, all these advantages make such a sensing technology very promising in the field of SCG monitoring. The details of the main studies that used FBGs for recording precordial vibrations are schematically reported in Table 4.

## 5. Conclusions

In the present review, we described wearable systems, signal processing techniques, and application scenarios for the recording of precordial vibrations. The aim was to go into detail about these three interrelated aspects that are fundamental in this research field and, if well-modulated, contribute to achieving a highly performant wearable system. Indeed, the use of wearables in this field is gaining increasing interest for the possibility to extract diagnostic indicators, thus enabling an early diagnosis of different types of cardiac dysfunctions. In this work, we focused on wearable systems for monitoring cardiac mechanics, and we identified three main approaches for monitoring precordial vibrations according to the measurand: acceleration (by accelerometers), angular velocity/rotation (by gyroscopes), and strain sensors (especially FBGs). For each technique, we gave details about the output signal, because their characteristic features are still not fully known. Moreover, we presented the sensors embedded in wearable systems for signal collection, the processing techniques explored for data analysis, and the main application scenarios in which the technique has been tested. 

According to the current state of the art, SCG monitoring using accelerometer-based wearables is the most well established technique. Several commercial or custom-made wearable systems integrating accelerometers have been tested for SCG recording, and many signal processing techniques have been explored for HR extraction, waveform annotation, and signal recognition/matching tasks. Moreover, different application scenarios in various conditions (e.g., at rest, during exercise) have been investigated. Several studies also focused on the use of this technique on HF patients, and its feasibility to assess the clinical status of these patients has been successfully demonstrated.

GCG has a shorter history than SCG, but it may provide additional understanding about the mechanical aspects of the cardiac cycle. The validity of SCG/GCG sensor fusion or combination has been assessed in the estimation of HR, HRV, CTIs, and annotation of waveforms. In addition, the GCG has been explored in various application modalities, including challenging dynamic conditions (e.g., during physical activity).

Lastly, FBG sensors for SCG recording are at an early stage; the analysis of strain-derived SCG is still limited to HR estimation, and no waveform analysis has been performed to identify fiducial points on this signal. However, FBG-based wearables are customizable, allowing a great freedom of design and adaptability to the application scenario. Furthermore, the immunity to electromagnetic interferences makes this technique advantageous to be used in harsh medical environments such as MRI. This application scenario has been promptly explored, although experimental protocols in the literature are usually limited to static conditions (i.e., quiet breathing and apnea in a resting position). 

To date, the use of these techniques is limited to the research field. However, they are all very promising tools to be introduced into clinical practice for different purposes. For instance, they could be used to complement ECG-derived information, for remote monitoring of patients, and for applications in harsh medical environments (e.g., MRI), where the use of ECG and electrical sensors is forbidden. The use of these techniques in a clinical scenario is still hindered by several limitations and open challenges, regardless of the specific technique considered. The first reason is that wearable devices are highly sensitive to motion and breathing-related artefacts. Hence, it is necessary to minimize the noise induced in the signal by motion artefacts that may cover the peaks and features of interest. In this specific application, movement is particularly critical because it can vary greatly with the sensor position and because precordial vibrations to be detected are of microscopic dimensions. Hence, noise removal is still an open challenge at both the hardware and the software design levels. This limitation may be overcome by performing spot acquisitions instead of a 24 h monitoring. Indeed, although these wearables cannot be used to monitor a person’s heart function during actual movement or physical activity (i.e., real dynamic conditions), they can still be useful to perform random acquisitions to monitor the subject at rest (e.g., during pre- and post-exercise resting). This would allow assessing the cardiac performance not only under controlled conditions and in a limited period of time (i.e., during medical examination), but also in daily living situations where the heart has been put under stress and its compliance can be evaluated. 

These techniques would be very advantageous and could open up new scenarios in clinical settings such as the possibility to monitor HR and the cardiac function during MRI examinations. Indeed, a correlation has been found between MR-quantified flow and function parameters and SCG energy levels or other features. These findings may encourage the development of an easy clinical test to identify potential valves flow abnormalities.

## Figures and Tables

**Table 2:** Details of the main studies that measured precordial vibrations using gyroscopes.

Paper	Recorded Signals	Reference Signals	Extracted Features/Parameters	Filtering Technique	Acquisition Device	Location of Device	Application Scenario	Public Database	Enrolled Individuals
Choudhary et al. 2020 [52]	SCG at 1 kHz	ECG, PPG	—Fiducial points (IM, AO, IC, AC, pAC, MO)	BP ^1^ filter (20–30 Hz)	PCB that integrates an accelerometer (ADXL335, ±3 g), a pre-amplifier, a Butterworth LP ^2^ filter (50 Hz), and a buffer	Lower sternum	During both normal breathing and apnea.	—	8 healthy male subjects
Khosrow-Khavar et al. 2015 [39]	SCG	ECG	—Fiducial points (IM, AO, AC)	HP ^3^ filter (0.5, 5, 10, 20, and 30 Hz)	Accelerometer (Brüel and Kjær model 4381)	Upper sternal border	The lower part of the body in supine position was placed in a negative pressure chamber from −20 to −50 mmHg in steps of −10 mmHg.	—	18 healthy subjects (15 male + 3 female)
Khosrow-Khavar et al. 2017 [40]	SCG	ECG	—Fiducial points (IM, AO, AC) —CTIs (LVET, PEP)	BP ^1^ filter (0.3–40 Hz)	Accelerometer (Brüel and Kjær model 4381, Nærum, Denmark)	Upper sternum	The lower part of the body in supine position was placed in a negative pressure chamber from −20 to −50 mmHg in steps of −10 mmHg.	—	LBNP ^4^ raining dataset: 48 subjects (32 male + 16 female) SFU_GYM ^5^ test dataset: 65 healthy subjects BGH ^6^ test dataset: 25 patients with a history of cardiac disease (12 female + 3 male) TC ^7^ test dataset: 15 healthy old subjects
Sørensen et al. 2018 [41]	SCG at 5 kHz	SCG (reference for the second heart sound), echocardiography, ECG	—Fiducial points (AO, AC, AS, MO, MC)	1st-order LP ^2^ Butterworth filter (90 Hz)	Accelerometer (Silicon Designs 1521)	Xiphoid process	Supine position while the ECG and SCG were simultaneously recorded pre, during, and post echography.	—	45 healthy subjects (male + female)
Hsu et al. 2020 [59]	SCG	ECG	—SCG biometric matching tasks	BP ^1^ (0.5 Hz–100 Hz) and 3rd-order Savitzky–Golay filter with a time interval of 0.01s with signal detrending.	—	—	—	PhysioNet CEBS ^8^ database	—
Lin et al. 2018 [51]	SCG at 400 Hz	ECG, echocardiography	—Fiducial points (LCV, SCV, AF, PF, MFA, MFE)	BP ^1^ filter (0.3–50 Hz)	3-axis accelerometer (LIS331DLH, da STMicro- electronics, Ginevra, Svizzera)	4 sensors placed at the 4 cardiac auscultation sites in correspondence with the mitral, tricuspid, aortic, and pulmonary valves	ECG and SCG were simultaneously collected, for each subject, in the supine position. Then, these signals were recorded during echocardiography.	—	25 healthy subjects (13 male + 12 female)
Zia et al. 2019 [60]	SCG	ECG, ICG	—Identification of consistent time features that co-vary with AO and PEP metrics	FIR filter (1–40 Hz) with kaiser window	3-axis accelerometer and gyroscope	Sternum	Standing, walking at 3 mph on a treadmill, exercise (squat) and post-exercise rest.	—	17 healthy subjects (10 male + 7 female)
Gamage et al. 2019 [43]	SCG at 10 kHz	—	—Cluster SCG events based on their morphology and group the clustered events with respect to lung volume phases and respiratory flow signals	BP ^1^ filter (0.5–40 Hz)	3-axis accelerometer (Model 356A32, PCB Piezotronics, Depew, NY)	4th intercostal space near the left lower sternal border	—	—	5 healthy male subjects
Taebi et al. 2018 [44]	SCG at 10 kHz	—	—Feature extraction during different lung phases —Cluster SCG events into classes of HLV ^12^ and LLV ^13^	LP ^2^ filter (100 Hz)	3-axis accelerometer (Model 356A32, PCB Piezotronics, Depew, NY)	4th intercostal space and left sternal border	Supine on a bed with the chest tilted at 45°.	—	7 healthy male subjects
Shafiq et al. 2016 [55]	SCG at 500 Hz	ECG	—Fiducial points (AO e AC)	5th-order Butterworth BP ^1^ filter (1–35 Hz)	Accelerometer	Xiphoid process	Supine position while breathing normally.	—	5 healthy subjects
Khosrow-Khavar et al. 2015 [42]	SCG	ECG	—Fiducial points (IM, AC)	5th-order LP ^2^ Butterworth filter (30 Hz)	Accelerometer (Brüel and Kjær model 4381, Nærum, Denmark)	—	The lower half of the body of the subject was placed in a sealed chamber in which the pressure was gradually reduced to -50 mmHg.	—	LBNP ^4^ training dataset: 18 healthy subjects (15 male + 3 female) SFU_GYM ^5^ test dataset: 67 healthy subjects (35 male + 32 female)
Wick et al. 2012 [56]	SCG at 1.2 kHz	ECG, echocardiography	—Fiducial points (AC) —CTIs (R-AC delay)	HP ^3^ filter (50 Hz).	Custom device integrating two 3-axis accelerometers (ADXL327, Analog Devices, Inc., Norwood, MA)	4th intercostal space	Echocardiography, ECG, and SCG data were simultaneously recorded using both the custom device and the ultrasound machine in static conditions	—	2 healthy subjects (1 male + 1 female)
Tavakolian et al. 2010 [27]	SCG at 2.5 kHz	ECG, ICG, suprasternal pulsed Doppler	—STI (LVET, PEP, and QS2) —Stroke volume estimation	—	Accelerometer (Model 393C, PCB Piezotronics)	Midline of the sternum with the lower edge of the sensor on the xiphoid process	Suprasternal Doppler, SCG, ECG, and ICG were simultaneously recorded. For stroke volume estimation, the signal acquisition was conducted in two separate sessions at least a day apart. The signal from the first session was used for training and the second day for testing.	—	24 subjects (21 male + 3 female): 20 healthy subjects + 4 patients of the BGH ^6^ who had a history of heart attack and very low ejection fraction.
Choudhary et al. 2020 [46]	SCG at 5 kHz	—	—Fiducial points (AO)	—	Custom device integrating an accelerometer (ADXL335, ±3 g)	Xiphoid process	Under both normal breathing and apnea in static conditions. The test was repeated in supine position during normal breathing and apnea, while sitting and standing, and during post-exercise recovery.	Test on CEBS ^8^ database	5 healthy male subjects + 20 healthy subjects from CEBS ^8^ database
Mora et al. 2020 [45]	SCG (SCG-1: 100 Hz; SCG-2: 5 kHz)	ECG	—Template generation	BP ^1^ FIR ^15^ filter (2–14 Hz)	3-axis accelerometer (ADXL 355 from Analog Devices, Inc.)	Xiphoid process for datasets SCG-1 and SCG-2	2 datasets of SCG and ECG signals. SCG-1: SCG recorded on 13 healthy volunteers in sitting position. SCG-2: public dataset	Dataset SCG-2: dataset CEBS	Dataset SCG-1: 13 healthy subjects Dataset SCG-2: 20 healthy subjects
Choudhary et al. 2019 [69]	SCG at 5 kHz	—	—Fiducial points (AO)	5th-order median filter	—	—	—	CEBS ^8^ database	—
Hsu et al. 2021 [47]	SCG at 150Hz	Blood pressure	—HR estimation	3rd-order Savitzky–Golay filter of 100 ms span, 6th-order LP ^2^ Butterworth filter (35 Hz), and interpolation with spline cubic curves at 750 Hz	3-axis accelerometer (MPU-6050)	Sternum	During both static (sitting) and dynamic (walking) conditions.	—	20 healthy subjects (14 male + 6 female)
Lin et al. 2020 [58]	SCG at 5 kHz	ECG	—HR estimation	—	—	—	—	CEBS ^8^ database	20 healthy subjects (12 male + 8 female)
Garcia-Gonzales et al. 2013 [61]	SCG at 5 kHz	ECG	—HR estimation	4th-order BP ^1^ Butterworth filter (5–30 Hz)	3-axis accelerometer (LIS344ALH, ST Microelectronics)	—	During static condition (supine position on a single bed). After 5 min of basal state, subjects listened to music for ~50 min. Finally, all subjects were monitored for 5 min after the music ended.	—	17 healthy subjects (11 male + 6 females).
Dinh et al. 2011 [53]	SCG at 400 Hz	ECG	—HR estimation	2 stages of LP ^2^ filtering (40 Hz)	PCB with a 3-axis accelerometer (MMA7260QT, made by Freescale).	—	Pre-exercise (in sitting, standing, and supine position), during exercise (walking), post-exercise (standing)	—	1 healthy subject
Choudhary et al. 2021 [64]	SCG (CEBS database: 5 kHz; private database: 1 kHz)	ECG	—Fiducial points (AO) —HRV estimation	—	—	—	—	CEBS ^8^ database + private database ^14^	CEBS ^8^ database: 20 healthy subjects Private database ^14^: 3 healthy male subjects
Ramos-Castro et al. 2012 [48]	SCG at 1 kHz	ECG	—HR estimation	4th-order Butterworth BP ^1^ filter (6–25 Hz)	In the first group, a 3-axis accelerometer (ADXL330, Analog Devices) with a low-pass frequency of 100 Hz was used, while, in the second group, an iPhone 4 was used.	Sternum	In supine position	—	12 healthy subjects
Tadi et al. 2015 [57]	SCG at 800 Hz	ECG	—HRV estimation	BP ^1^ filter (4–50 Hz) with moving average filter (window duration of 10 and 20 ms)	3-axis capacitive digital accelerometer (MMA8451Q from Freescale Semiconductor)	Sternum	Supine position on a bed	—	20 healthy male subjects
Shandhi et al. 2022 [66]	SCG at 500 Hz	ECG	—Estimate changes in PAM ^9^ and PCWP ^10^	BP ^1^ filter (1–40 Hz)	Custom-built wearable patch embedding a PCB with a 3-axis accelerometer (BMA280 from Bosch Sensortec GmbH, Reutlingen, Germany)	Middle of the sternum	During RHC ^11^ procedure	—	20 patients with HF
Chen et al. 2020 [65]	SCG at 1 kHz	ECG	—Cluster waveforms based on similar morphology —Template generation	HP ^3^ filter (40 Hz)	Accelerometer	4 sensors placed at the 4 cardiac auscultation sites in correspondence with the mitral, tricuspid, aortic, and pulmonary valves	Supine position at rest	—	16 total subjects: 8 healthy subjects + 8 patients with HF

^1^ BP: bandpass. ^2^ LP: low pass. ^3^ HP: high pass. ^4^ LBNP: low body negative pressure. ^5^ SFU_GYM: Simon Fraser University Gymnasium. ^6^ BGH: Burnaby General Hospital. ^7^ TC: Terminal Club. ^8^ CEBS: “Combined measurements of ECG, Breathing, and Seismocardiogram” database. The dataset contains 1 h ECG, respiration, and SCG data of 20 subjects (12 male + eight female) in supine position, collected at a frequency of 5 kHz. For the central 50 min, the subjects listened to classical music at a frequency of 5 kHz. The models were trained on the first 5 min of SCG, and the identification of fiducial points was performed on the last 5 min of SCG. A Biopac MP36 DAQ (Santa Barbara, CA, USA) was used to record the ECG with electrodes (3M Red Dot 2560). One channel of the Biopac MP36 DAQ + a piezoresistive chest band (SS5LB sensor by Biopac, Santa Barbara, CA, USA) were used to collect the respiratory signal. The Biopac MP36 DAQ + a three-axis accelerometer (LIS344ALH, ST Microelectronics) were used to acquire the SCG signal. The dataset is available at https://archive.physionet.org/physiobank/database/cebsdb/ (accessed on 5 May 2022). ^9^ PAM: pulmonary artery mean pressure. ^10^ PCWP: pulmonary capillary wedge pressure. ^11^ RHC: right-heart catheterization. ^12^ HLV: high lung volume. ^13^ LLV: low lung volume. ^14^ The private database contains 15 multichannel SCG signals recorded from three healthy male subjects in five different sessions. These sessions involved various physiological modulations and postures: (i) supine position with normal breathing for 6 min, (ii) supine position with hold or stopped breathing for 40 s, (iii) sitting for 2 min, (iv) standing for 2 min, and (v) exercise recovery. The exercise recovery included rope-skipping (1 min) and a plank exercise (30 s) followed by a recovery period of 20 s. The signal acquisition was accomplished using a small custom wearable electronic device. The system consisted of a miniaturized MEMS accelerometer (ADXL335, ±3 g), pre-amplifier, Butterworth LP filter (50 Hz), buffer, data acquisition system (Biopac MP150), and PC with the AcqKnowledge interfacing software. Signals were sampled at 1 kHz. ^15^ FIR: finite impulse response.

**Table 3:** Details of the main studies that measured precordial vibrations using gyroscopes.

Paper	Recorded Signals	Reference Signals	Extracted Features/Parameters	Filtering Technique	Acquisition Device	Location of Device	Application Scenario	Public Database	Enrolled Individuals
Yang et al. 2017 [63]	GCG and first derivative of GCG (DGCG) at 256 Hz	ECG, ICG, SCG	—Fiducial points (IM, A0, AC) —CTIs (LVET, PEP)	BP ^1^ Butterworth filter (0.8–25 Hz)	IMU (Shimmer 3 from Shimmer Sensing): 3-axis accelerometer (Kionix KXRB5-2042, Kionix, Inc.) + 3-axis gyroscope (Invensense MPU9150, Invensense, Inc., San Jose, CA, USA).	Along the second and third rib at the middle of the sternum	Sitting on a chair pre-exercise, steps climbing and resting post-exercise	—	5 healthy subjects (3 male + 2 female)
D’Mello et al. 2019 [11]	SCG combined with GCG (VCG ^16^) at 250 Hz	ECG	—Fiducial points (AO) —HR estimation	HP ^3^ brick wall filter (0.4 Hz).	InvenSense Motion Processing UnitTM 9250 consisting of a MEMS gyroscope and accelerometer	Xiphoid process	Resting supine, high intensity physical exercise and resting post-exercise.	—	25 healthy male subjects
Dehkordi et al. 2020 [76]	GCG standalone and combined with SCG at 1 kHz	SCG, ECG, ICG, echocardiogram	—Fiducial points (AO, AC, MO, MC) —CTIs (EMD, PEP, ST, Q-MO, LVET, IVCT, IVRT) —Tei index	—	IMU (ASC GmbH, ASC IMU 7.002LN.0750, Pfaffenhofen, Germany): low-noise 3-axis MEMS joint accelerometer-gyroscope sensor	—	—	—	50 healthy subjects (23 male + 27 female)
Tadi et al. 2017 [72]	GCG at 800 Hz	SCG, ECG, echocardiogram	—Fiducial points (AVO, AVC, MVO, MVC) —CTIs (LVET, PEP, QS2, IVRT, IVCT, Q-SPV, Q-DPV)	4th-order BP ^1^ Butterworth IIR ^17^ filter (1–20 Hz)	Custom-made IMU: 3-axis low-power capacitive digital accelerometer (Freescale Semiconductor, MMA8451Q, Austin, TX, USA) + low-power low-noise 3-axis gyroscope (Maxim Integrated, MAX21000, San Jose, CA, USA)	Middle of the sternum	Lying down in the supine position with the upper body slightly tilted.	—	9 healthy male subjects
Kaisti et al. 2019 [75]	GCG combined with SCG at 800 Hz	ECG	—HR estimation	Filtered with a 3rd-order BP ^1^ Butterworth IIR ^17^ filter (0.5–20 Hz)	IMU: 3-axis capacitive digital accelerometer (Freescale Semiconductor, MMA8451Q, Austin, TX, USA) + 3-axis gyroscope (Maxim Integrated, MAX21000, San Jose, CA, USA)	Sternum	Lying either in the supine position or on left or right side.	—	*Dataset 1:* 29 healthy male subjects. *Dataset 2:* 12 patients with coronary artery disease (10 male + 2 female)
Sieciński et al. 2020 [77]	GCG and SCG at 800 Hz	ECG	—HRV analysis	3rd-order Butterworth BP ^1^ filter (4–50 Hz) with zero-phase FIR moving average filter with the window width of 15 ms; to align the baseline with zero, the signals resulted from beat detection were filtered with the 3rd-order BP ^1^ Butterworth filter (1 Hz and 40 Hz)	—	—	—	Mechanocardiograms with ECG Reference data set ^18^	—

^1^ BP: bandpass. ^3^ HP: high pass. ^16^ VCG: vibrational cardiography. ^17^ IIR: infinite impulse response. ^18^ The “Mechanocardiograms with ECG Reference” dataset by Kaisti et al. is publicly available from the IEEE DataPort data repository. This dataset consists of 29 mechanocardiogram recordings with ECG reference. The signals were recorded from 29 healthy male subjects while in supine position. All data were recorded with sensors attached to the sternum using double-sided tape and a frequency of 800 Hz. Mechanocardigrams include accelerometer signals (SCG) and gyroscope signals (GCG) recorded using a three-axis capacitive digital accelerometer (MMA8451Q from Freescale Semiconductor, Austin, TX, USA) and a three-axis MAX21000 gyroscope (Maxim Integrated, San Jose, CA, USA), respectively. ECG signals were collected using ADS1293 from Texas Instruments.

**Table 4:** Details of the main studies that used FBGs to measure precordial vibrations.

Paper	Recorded Signals	Reference Signals	Extracted Features/Parameters	Filtering Technique	Acquisition Device	Location of Device	Application Scenario	Public Database	Enrolled Individuals
Lo Presti et al. 2019 [93]	SCG	PPG	—HR estimation	2nd-order BP ^1^ Butterworth filter (0.8–2 Hz)	A commercial FBG (λ_*B*_ of 1547 nm, grating length of 10 mm, and reflectivity of 90%; AtGrating Technologies) encapsulated into a frame of Dragon skin^®^20 silicone rubber (Smooth-On, Inc., Macungie, PA, USA) of dimensions 90 mm × 24 mm × 1 mm.	Lower thorax	Each volunteer was asked to perform two tests consisting of a stage during both quiet breathing and apnea	—	2 healthy subjects (1 male + 1 female)
Chethana et al. 2017 [96]	SCG	Stethoscope	—HR estimation (average HR per minute)	HP ^3^ filter (0.5 Hz)	The sensor is made of a cone-shaped structure whose end is made up of polyvinyl chloride, a micrometer, and a flexible silicon diaphragm. A 9/125 μm diameter germania-doped photosensitive silica fiber was used in the fabrication of FBG sensors of 3 mm gauge length. The fabricated FBG sensor was tightly bonded across the diaphragm using a thin layer of cyanoacrylate adhesive.	Around 2nd and 3rd interspace of pulmonic area	Under different breathing conditions (slow, automatic inhalation and exhalation, forced inhalation and exhalation)	—	4 healthy subjects (2 male + 2 female)
Nedoma et al. 2019 [88]	SCG at 1 kHz	ECG	—HR estimation	3rd-order Butterworth BP ^1^ filter (5–20 Hz)	The sensor (dimensions 30 × 10 × 0.8 mm and weight 2 g) is made of a fiberglass structure (type Epikote Resin MGS LR 285 and Curing Agent MGS LH 285) of length 1.8 mm, which encapsulates a Bragg grating with a λ_*B*_ of 1550.218 nm. The sensor was designed as part of a contact elastic belt.	Around the pulmonic area near to the heart	During MRI procedures	—	10 healthy subjects (6 male + 4 female)
Nedoma et al. 2017 [92]	SCG at 300 Hz	—	—HR estimation	BP ^1^ Butterworth IIR ^17^ -filter (1–5 Hz).	The measuring probe consists of the uniform FBG with polyamide protection with λ_*B*_ of 1554.1207 nm. The width of the reflecting spectrum was 2.3241 nm, and reflectivity was 95.7%. It was encapsulated into a PDMS polymer of rectangular shape.	Left side of the upper chest in an area of the heart	standing, sitting and supine	—	5 healthy subjects
Tavares et al. 2022 [95]	SCG at 1 kHz	ECG	—HR estimation	BP ^1^ filter (0.8–2.0 Hz)	The sensor consists of an elastic material (*Flexible*, Fish box mini model, Avistron, Bergheim, Germany) printed by a 3D printer (Ultimaker 3D Extended, Ultimaker, Utrecht, Netherlands) and a single optical fiber with a single FBG.	Left side of the chest	During apnea and normal breathing while lying down on a physiotherapy bed	—	3 healthy subjects

^1^ BP: bandpass. ^3^ HP: high pass. ^17^ IIR: infinite impulse response.

## Data Availability

Not applicable.

## Supplementary Materials

The following supporting information can be downloaded at: https://www.mdpi.com/article/10.3390/s22155805/s1, Section S1: Accelerometer working principle; Section S2: Gyroscope working principle; Section S3: FBG working principle.
