# Supplementary material for: Precordial Vibrations: A Review of Wearable Systems, Signal Processing Techniques, and Main Applications
Source: Sensors (Basel). 2022 Aug 3;22(15):5805. doi: 10.3390/s22155805 (PMC9370957; doi:10.3390/s22155805)
Supplement: Supplementary file 1 [file sensors-22-05805-s001.zip › sensors-1820291-supplementary.pdf]

sensors MDPI

Supplementary Materials

1. Accelerometer working principle

The accelerometer can be modeled as a mass-spring-dampen system: if a force is applied to the system, the outer shell accelerates while the internal mass (i.e., m) tends to remain stationary due to the principle of inertia and therefore the spring stretches. The stiffness of the spring, (i.e., k_{el}) tends to restore m back to the equilibrium point, while internal frictional damping (i.e., b) opposes any displacement from the equilibrium point. Such a model is represented by the following equation:

$$m \cdot \ddot{x_1} + b \cdot \dot{x_1} + k \cdot x_1 = 0 \quad (1)$$

with a damping factor $\zeta = \frac{b}{2m\omega_n}$ and natural pulsation $\omega_n = \sqrt{\frac{k}{m}}$.

In the SCG recording application, the accelerometer is mechanically coupled to the thorax and as the chest wall surface moves, the mass of the accelerometer moves with an inertial response. Considering x_1 the position of m and x_2 the displacement of the chest wall and of the outer shell of the accelerometer, this mechanical model can be represented as follows:

$$m \cdot \ddot{x_1} + b \cdot (\dot{x_1} - \dot{x_2}) + k \cdot (x_1 - x_2) = 0 \quad (2)$$

With $(x_1 - x_2)$ iand its first derivative being respectively the relative position and relative motion of the internal mass of the accelerometer with respect to the outer sensor shell.

2. Gyroscope working principle

MEMS gyroscopes are based on a vibrating mechanical element as a sensing element anchored to a rotating frame and on an energy transfer between two vibrational modes caused by the apparent acceleration of Coriolis $a_c = 2v \cdot \Omega$. Indeed, MEMS gyroscopes can be modeled as mass-spring-dampen systems with a mass that moves along two orthogonal mechanical excitation modes. The in-plane rotation of a rigid body in a three-dimensional space can be described using Euler angles (φ', ϑ', ψ'). Angular velocities ω_x , ω_y and ω_z generated by rotation along the x, y, and z axis are related to Euler angles as follows:

$$\begin{bmatrix} \varphi' \\ \vartheta' \\ \psi' \end{bmatrix} = \begin{bmatrix} 1 & \sin\varphi \tan\vartheta & \cos\varphi \tan\vartheta \\ 0 & \cos\varphi & -\sin\varphi \\ 0 & \sin\varphi \cos\vartheta & \cos\varphi \cos\vartheta \end{bmatrix} \begin{bmatrix} \omega_x \\ \omega_y \\ \omega_z \end{bmatrix} \quad (3)$$

According to (4), estimation of the change in the thorax angle using only a gyroscope implies integral calculation. In this process, integration of the change in the thorax angle and sensor errors may cause a divergent output (i.e., sensor drift). For this reason, gyroscopes are preferably used in conjunction with a tri-axial accelerometer:

$$a = v' + (v \cdot \omega) + R^T \cdot g \quad (4)$$

$$\begin{bmatrix} a_x \\ a_y \\ a_z \end{bmatrix} = \begin{bmatrix} v'_x \\ v'_y \\ v'_z \end{bmatrix} + \begin{bmatrix} 0 & v_z & -v_z \\ -v_z & 0 & v_x \\ v_z & v_x & 0 \end{bmatrix} \begin{bmatrix} \omega_x \\ \omega_y \\ \omega_z \end{bmatrix} + R^T \begin{bmatrix} 0 \\ 0 \\ -9.81 \end{bmatrix} \quad (5)$$

Where vectors ν' and ω' are the linear acceleration and angular velocity respectively and R is the rotated matrix defined as:

$$R = \begin{bmatrix} \cos\vartheta\cos\varphi & \sin\psi\sin\vartheta\cos\varphi - \cos\psi\sin\varphi & \cos\psi\sin\vartheta\cos\varphi + \sin\psi\sin\varphi \\ \cos\vartheta\sin\varphi & \sin\psi\sin\vartheta\sin\varphi + \cos\psi\cos\varphi & \cos\psi\sin\vartheta\sin\varphi - \sin\psi\cos\varphi \\ -\sin\vartheta & \cos\vartheta\sin\psi & \cos\vartheta\cos\psi \end{bmatrix} \quad (6)$$

Hence, equation (5) can be rearranged as:

$$\begin{bmatrix} a_x \\ a_y \\ a_z \end{bmatrix} = \begin{bmatrix} \nu_x' \\ \nu_y' \\ \nu_z' \end{bmatrix} + \begin{bmatrix} 0 & \nu_z & -\nu_z \\ -\nu_z & 0 & \nu_x \\ \nu_z & \nu_x & 0 \end{bmatrix} \begin{bmatrix} \omega_x \\ \omega_y \\ \omega_z \end{bmatrix} + \begin{bmatrix} \sin\vartheta \\ \cos\vartheta\sin\psi \\ -9.81\cos\vartheta\cos\psi \end{bmatrix} \quad (7)$$

Where ϑ and ψ denote the change in the angle of the thorax caused by cardiac vibrations in absence of linear velocity, respiration artefact and rapid body movement.

3. FBG working principle

FBGs are manufactured by photo-etching a permanent periodic variation of the refractive index into the core of a special type of optical fiber. This modulation, along the beam propagation direction, is realized by exposing a segment of a few millimeters of the optical fiber to a periodic pattern of an intense ultra-violet (UV) source (e.g., UV laser). Usually, a germanium-doped silica fiber is used in the manufacture of FBGs. The germanium-doped fiber is photosensitive, so the refractive index of the core changes with exposure to the UV light and the amount of change depends on the intensity and duration of the exposure as well as on the photosensitivity of the fiber.

The back reflected spectrum is centered around the so-called Bragg wavelength, λ_B, which depends on the effective refractive index of the fiber core and on the grating spatial period, Λ:

$$\lambda_B = 2\Lambda\, \eta_{eff} \quad (8)$$

Both the terms in the Bragg condition are sensitive to ε and temperature (T), thus the use of a proper configuration and design allows the estimation of these parameters by monitoring changes of λB. Indeed, when an FBG is exposed to ε and T changes, a variation in Λ and η_{eff} occurs causing a shift of λ_B ($\Delta\lambda_B$). Differentiating the Bragg condition in equation 8 and neglecting higher order terms, the $\Delta\lambda_B$ is given by:

$$\Delta\lambda_B = 2\left(\Lambda\frac{\delta\eta_{eff}}{\delta z} + \eta_{eff}\frac{\delta\Lambda}{\delta z}\right)\Delta z + 2\left(\Lambda\frac{\delta\eta_{eff}}{\delta T} + \eta_{eff}\frac{\delta\Lambda}{\delta T}\right)\Delta T = \Delta\lambda_B{}^{mech} + \Delta\lambda_B{}^{therm} \quad (9)$$

Where Δz is the change in grating length and ΔT is the change in grating T. In the mechanical contribution, the first addend represents the photo-elastic effect and the second one expresses the variation of the geometry, while in the thermal contribution the first addend is the thermo-optic effect, and the second addend is related to thermal expansion. Therefore, wavelength sensitivity of FBGs is governed by the elastic, elasto-optic and thermo-optic properties. Hence, the first term in equation 9 represents the ε effect on an optical fiber ($\Delta\lambda_B{}^{mech}$) and the second term represents the effect of T ($\Delta\lambda_B{}^{therm}$). These terms can be expressed as:

$$\Delta\lambda_B{}^{therm} = \lambda_B\, S_T\, \Delta T \quad (10)$$

$$\Delta\lambda_B{}^{mech} = \lambda_B\, S_\varepsilon\, \varepsilon \quad (11)$$

Where S_T is the sensitivity to T changes and S_ε is the sensitivity to ε which are determined by means of a calibration process. Hence, FBG sensors are intrinsically sensitive to both these parameters, but several strain-

temperature discrimination techniques and particular sensor encapsulation packages have enabled to develop sensors that are selectively sensitive to only one of the two physical quantities. When an FBG sensor is able to measure a single extrinsic property of a system, it is called a "single parameter" sensor.

During cardiac monitoring applications, the FBG should be highly sensitive to ε and the influence of T should be considered negligible. It can be assumed that during the time span of cardiac monitoring, ΔT will never be greater than 0.03 °C causing a maximum $\Delta\lambda_B \approx 27 \times 10^{-3}$ pm. For this reason, temperature influence on λ_B can be assumed negligible compared to strain effects on the grating. Under such a hypothesis, the strain effect on an FBG sensor can be expressed as:

$$\Delta\lambda_B{}^{mech} = \lambda_B\, S_\varepsilon\, \varepsilon = \lambda_B \left\{1 - \frac{{\eta_{eff}}^2}{2}[P_{12} - \nu(P_{11} - P_{12})]\right\} \varepsilon = \lambda_B\, (1 - \rho_\varepsilon)\, \varepsilon \tag{12}$$

$$\frac{\Delta\lambda_B{}^{mech}}{\lambda_B} = (1 - \rho_\varepsilon)\, \varepsilon \tag{13}$$

where P_{11} and P_{12} are the silica photo-elastic tensor components, ν is the Poisson's ratio, ε is the strain change, and ρ_ε is the stress-optic coefficient. For a germanium-doped core and a wavelength of 1550 nm, the strain change of a silica fiber is approximately 1.20 pm·$(\mu\varepsilon)^{-1}$.
